# An avian influenza H7 DNA priming vaccine is safe and immunogenic in a randomized phase I clinical trial

**DOI:** 10.1038/s41541-017-0016-6

**Published:** 2017-06-01

**Authors:** Adam D. DeZure, Emily E. Coates, Zonghui Hu, Galina V. Yamshchikov, Kathryn L. Zephir, Mary E. Enama, Sarah H. Plummer, Ingelise J. Gordon, Florence Kaltovich, Sarah Andrews, Adrian McDermott, Michelle C. Crank, Richard A Koup, Richard M. Schwartz, Robert T. Bailer, Xiangjie Sun, John R. Mascola, Terrence M. Tumpey, Barney S. Graham, Julie E. Ledgerwood

**Affiliations:** 1 Vaccine Research Center (VRC), National Institute of Allergy and Infectious Diseases, National Institutes of Health, Bethesda, MD 20892 USA; 2 Biostatistics Research Branch, National Institute of Allergy and Infectious Diseases, National Institutes of Health, Bethesda, MD 20892 USA; 3 Influenza Division, National Center for Immunization and Respiratory Diseases, US Centers for Disease Control and Prevention, Atlanta, GA USA; 4 Present Address: Synlogic, Cambridge, MA 02139 USA

## Abstract

A novel avian influenza subtype, A/H7N9, emerged in 2013 and represents a public health threat with pandemic potential. We have previously shown that DNA vaccine priming increases the magnitude and quality of antibody responses to H5N1 monovalent inactivated boost. We now report the safety and immunogenicity of a H7 DNA-H7N9 monovalent inactivated vaccine prime-boost regimen. In this Phase 1, open label, randomized clinical trial, we evaluated three H7N9 vaccination regimens in healthy adults, with a prime-boost interval of 16 weeks. Group 1 received H7 DNA vaccine prime and H7N9 monovalent inactivated vaccine boost. Group 2 received H7 DNA and H7N9 monovalent inactivated vaccine as a prime and H7N9 monovalent inactivated vaccine as a boost. Group 3 received H7N9 monovalent inactivated vaccine in a homologous prime-boost regimen. Overall, 30 individuals between 20 to 60 years old enrolled and 28 completed both vaccinations. All injections were well tolerated with no serious adverse events. 2 weeks post-boost, 50% of Group 1 and 33% of Group 2 achieved a HAI titer ≥1:40 compared with 11% of Group 3. Also, at least a fourfold increase in neutralizing antibody responses was seen in 90% of Group 1, 100% of Group 2, and 78% of Group 3 subjects. Peak neutralizing antibody geometric mean titers were significantly greater for Group 1 (GMT = 440.61, *p* < 0.05) and Group 2 (GMT = 331, *p* = 0.02) when compared with Group 3 (GMT = 86.11). A novel H7 DNA vaccine was safe, well-tolerated, and immunogenic when boosted with H7N9 monovalent inactivated vaccine, while priming for higher HAI and neutralizing antibody titers than H7N9 monovalent inactivated vaccine alone.

## Avian influenza: A vaccine for a deadly emergent strain

A vaccine candidate to treat a deadly subtype of avian influenza was shown to induce protective antibodies in initial clinical trials. As of March 2017, avian influenza strain A/H7N9 has killed 497 people since 2013, with 1349 confirmed cases. Julie Ledgerwood and her team from the United States’ National Institutes of Health in collaboration with colleagues at the Centers for Disease Control and Prevention tested their two-stage vaccine protocol in humans, showing it to be effective and safe. The vaccine consists of an initial injection of viral DNA, which ‘primes’ the immune system to the pathogen, followed by a follow-up injection of an inactivated purified viral protein, which further boosts the host’s production of protective antibodies. The study shows the viability of this vaccine regimen and suggests further investigation into its appropriateness for treating avian influenza in humans.

## Introduction

In February 2013, a novel avian influenza subtype, A/H7N9, appeared in China resulting in severe lower respiratory tract infections in humans.^1^ H7N9 is a low pathogenic avian influenza A virus that emerged in poultry following a likely triple reassortment event among avian influenza A viruses circulating in Asian birds.^2^ H7N9 does not cause an identifiable illness in poultry and had not been documented in humans prior to 2013 (refs. 3, 4). As of March 22, 2017, 1349 laboratory confirmed cases of H7N9 with 497 deaths (36.8%) have been reported.^5^

Pandemic influenza can arise from a reassortment event in which two or more influenza viruses exchange genetic material and/or via direct spread from animals to humans of an influenza virus that has adapted to spread in humans.^6, 7^ While sustained human-to-human transmission of H7N9 has not been reported, several factors suggest H7N9 has pandemic potential. These include the absence of baseline immunity to H7N9 in humans;^3, 8^ presence of genetic markers associated with human adaptation such as Q226L in the HA gene and an E627K substitution in PB2 (ref. 4); evidence of transmission through direct contact and airborne exposure in a ferret model;^9^ and evidence that H7N9 not only can replicate in human airway epithelial cells, but also achieves higher peak viral titers in human bronchus and lung than other avian influenza viruses such as H5N1 (refs. 10, 11).

Protection against influenza is primarily antibody mediated. Humoral responses to candidate influenza vaccines are evaluated by the hemagglutination inhibition (HAI) assay and by measurement of neutralizing antibodies. HAI evaluates antibody responses to strain-specific antigenic sites in the head region of influenza hemagglutinin (HA) where titers of ≥1:40 are traditionally reported as a correlate of protection from seasonal influenza.^12^ Neutralizing antibody assays provide an alternative and functional assessment of antibody responses that are capable of inhibiting infection of cells.

There are currently no Food and Drug Administration approved vaccines for the prevention of H7N9. H7 HA may be intrinsically poorly immunogenic and candidate H7 vaccines have historically generated limited immune responses.^13–15^ While poorly immunogenic on their own, live attenuated influenza vaccines (LAIV) for H7N7 have been shown to effectively prime the immune system for an H7N7 inactivated influenza boost.^16^ Similarly, a candidate H7N9 LAIV primed for robust antibody responses following an antigenically matched inactivated boost.^17^

DNA vaccines induce both cellular and humoral immunity, can be manufactured rapidly, and have been shown to be safe and immunogenic in vaccine regimens against influenza, HIV, Ebola, SARS, and West Nile Virus.^18–21^ We have previously shown that a DNA vaccine encoding H5 increased the magnitude and quality of antibody responses when used as a priming vaccine prior to H5N1 monovalent inactivated boost.^22–24^ A DNA vaccine encoding H7 was subsequently developed and shown to provide protection from H7N9 lethal challenge in pre-clinical testing in mice (unpublished data). Herein, we report on the safety and immunogenicity of a prime-boost regimen consisting of H7 DNA followed by a H7N9 monovalent inactivated vaccine (MIV) boost. In a prior phase 1 clinical trial, H7N9 MIV was found to be safe but poorly immunogenic in the absence of adjuvant.^25^ We consequently evaluated the ability of this prime-boost regimen to improve H7-specific HAI titers and neutralizing antibody responses.

## Results

**Fig. 1:**
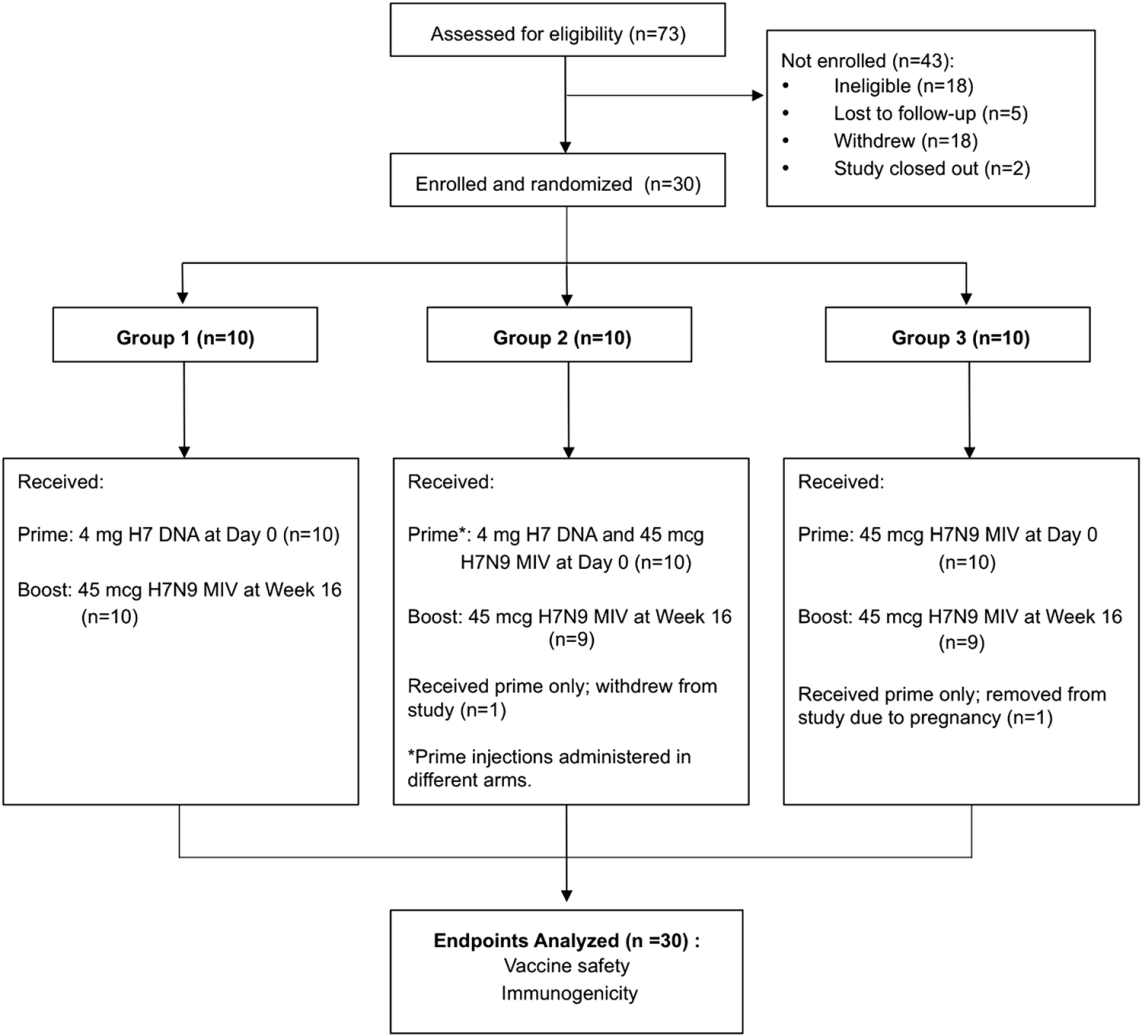
CONSORT flow diagram of the trial. Consolidated standards of reporting trials (CONSORT) diagram delineates study enrollment of 30 subjects who were randomized to three study groups

Thirty healthy individuals were enrolled between January 20, 2015 and March 11, 2015 with the last subject visit occurring on September 21, 2015 (Fig. 1). Demographic characteristics were similar between groups (Supplemental Table 1). There were no vaccine-related serious adverse events and the vaccines were well tolerated. When present, reactogenicity was mild to moderate in severity (Supplemental Tables 2A and 2B).

**Fig. 2:**
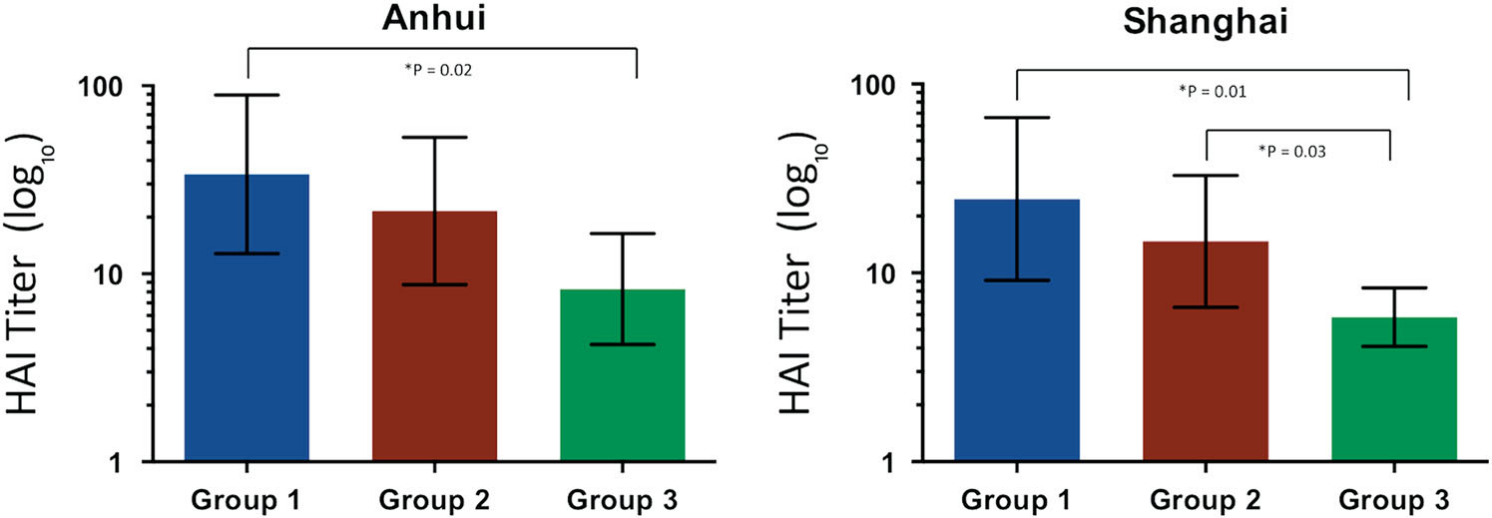
Induction of H7-specific antibodies following three different prime-boost regimens. *HAI* = hemagglutination inhibition assay. Geometric mean titers and 95% confidence intervals are shown 2 weeks following H7N9 boost vaccination. Group 1 received H7 DNA at day 0 and H7N9 MIV at week 16. Group 2 received both H7 DNA and H7N9 MIV prime at day 0 and H7N9 MIV at week 16. Group 3 received H7N9 MIV at day 0 and at week 16. Groups with statistically significant responses are marked with corresponding *p*-values (Student’s *t*-test in log measurements)

All subjects had HAI titers<1:10 at baseline to two related antigenic strains of H7N9, A/Anhui/1/2013 and A/Shanghai/2/2013, consistent with an H7 naive population at baseline (Fig. S1). H7-specific antibody responses were assessed at week 18 (2 weeks following the 16 week H7N9 MIV boost). One-way analysis of variance (ANOVA) with post hoc *t*-test indicated statistically significant differences in HAI titer across the three groups at week 18 (*p* = 0.03 for the Anhui antigen and *p* = 0.02 for the Shanghui antigen). The magnitude and frequency of HAI responses was greater in group 1 (DNA prime-MIV boost) and group 2 (DNA/MIV prime-MIV boost) than in group 3 (MIV prime-MIV boost), indicating H7 DNA primed for a better HAI response than H7N9 MIV (Fig. 2, Table 1). Two weeks following the boost, peak GMT were higher in those receiving H7 DNA prime (groups 1 and 2) rather than H7N9 MIV prime alone (group 3), and this difference reached significance between groups 1 and 3 (33.8 vs. 8.3, *p* = 0.02) for the Anhui antigen and between groups 1 and 3 (24.6 vs. 5.8, *p* = .01) and groups 2 and 3 (14.7 vs. 5.8, *p* = 0.03) for the Shanghai antigen (Fig. 2). While higher titers were observed in group 1 compared to group 2, this difference was not significant for either antigen. Five of 10 individuals in group 1 achieved a positive response titer of ≥1:40 to either the Anhui or Shanghai antigen at 2 weeks post-boost while 3 of 9 individuals in group 2 and 1 of 9 individuals in group 3 achieved a positive HAI response ≥1:40 (Table 1). In all groups, peak HAI GMT was detected 2 weeks post-boost (Fig. 2, Table 1). In group 3 (MIV prime-MIV boost), peak HAI GMT was maintained until 12 weeks post-boost, although the titer was lower than both groups 1 and 2 (Fig. S1).

**Table 1:** HAI response by group assignment 2 weeks post boost vaccination (week 18) for Anhui and Shanghai H7N9 strains

	Group 1		Group 2		Group 3	
Antigen	Anhui	Shanghai	Anhui	Shanghai	Anhui	Shanghai
Two weeks post boost vaccination GMT (95% CI)	33.8 (12.8, 89.2)	24.6 (9.1, 66.5)	21.6 (8.8, 53.2)	14.7 (6.6, 32.8)	8.30 (4.2, 16.4)	5.8 (4.1, 8.3)
Individuals with post-boost titer ≥10 (95% CI)	80.0% 8/10 (0.44, 0.97)	70.0% 7/10 (0.35, 0.93)	77.8% 7/9 (0.4, 0.97)	66.7% 6/9 (0.3, 0.93)	33.3% 3/9 (0.07, 0.7)	11.0% 1/9 (0, 0.48)
Individuals with post-boost titer ≥40 (95% CI)	50.0% 5/10 (0.19, 0.81)	50.0% 5/10 (0.19, 0.81)	33.3% 3/9 (0.07, 0.7)	33.3% 3/9 (0.07, 0.7)	11.0% 1/9 (0, 0.48)	0.0% 0/9 (0, 0.34)

Group 1 received H7 DNA at day 0 and H7N9 at week 16. Group 2 received H7 DNA and H7N9 MIV at day 0 and H7N9 MIV at week 16. Group 3 received H7N9 MIV at day 0 and week 16. Subjects were negative for HAI to H7N9 at baseline

*GMT* = geometric mean titer

**Fig. 3:**
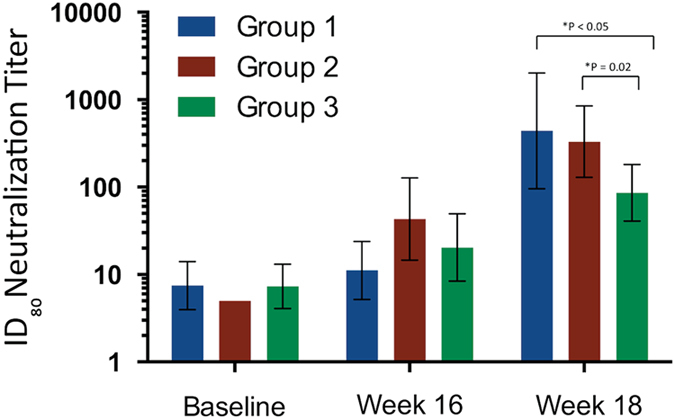
Neutralizing antibody responses (ID80) by group assignment. Geometric mean titers and 95% confidence intervals are shown at baseline, the day of boost, and 2 weeks following the H7N9 boost. Group 1 received H7 DNA at day 0 and H7N9 MIV at week 16. Group 2 received both H7 DNA and H7N9 MIV prime at day 0 and H7N9 MIV at week 16. Group 3 received H7N9 MIV at day 0 and at week 16. Groups with statistically significant responses are marked with corresponding *p*-values (Student’s *t*-test in log measurements)

Neutralizing antibody responses 2 weeks post-boost (week 18) were significantly different across the three groups based on one-way ANOVA (*p* = 0.01). Although the responses were similar between groups 1 and 2 (Fig. 3, Table 2), peak GMT were significantly greater for groups receiving the DNA prime, group 1 (440.6, *p* < 0.05) and group 2 (331.0, *p* = 0.02) when compared with Group 3 (86.1). A positive neutralizing antibody response was defined as a fourfold increase from baseline. Two weeks following the boost 90% of group 1, 100% of group 2, and 78% of group 3 achieved a positive neutralizing antibody response (Table 2) by this definition. Two weeks following the boost, the highest neutralizing antibody responses were identified in subjects who also achieved HAI titers ≥40.

**Table 2:** Neutralizing antibody responses by group assignment

	Group 1	Group 2	Group 3
Baseline GMT (95% CI)	7.5 (4.0, 14.0)	5 (5.0, 5.0)	7.32 (4.1, 13.2)
Week 16, day of vaccine boost GMT (95% CI)	11.1 (5.2, 23.8)	43.0 (14.6, 126.9)	20.4 (8.4, 49.4)
Week 18, 2 weeks post boost GMT (95% CI)	440.6 (95.9, 2024.0)	331.0 (128.9, 850.0)	86.1 (40.9, 181.5)
Positive response at week 18 (95% CI)	90% 9/10 (55, 100)	100% 9/9 (66, 100)	78% 7/ 9 (40, 97)

Geometric mean titers are shown at baseline, the time of H7N9 MIV boost and 2 weeks post-boost

In the above analyses on the magnitude of response, comparisons between two groups were based on two-sample *t*-tests on the log transformed data.Wilcoxon rank-sum tests were also performed, and they led to the same conclusions.

## Discussion

Influenza A/H7N9 is a public health threat for which a preventive vaccine may be needed to rapidly respond to a pandemic. In this phase 1 clinical trial, a novel H7 DNA vaccine was safe, well-tolerated and provided robust immunogenicity as a priming vaccine when boosted with H7N9 MIV. Given the absence of baseline immunity to H7 in the human population and the weak immunogenicity of H7, it is anticipated that H7N9 vaccine regimens will require more than one injection.^14, 26, 27^ Our results are consistent with this assertion. The DNA vaccine may provide an option for priming the immune system to improve the overall response to a heterologous booster vaccination, providing greater magnitude of antibody responses than with either vaccine alone.^27–30^ This may occur because DNA vaccines prime different populations of T cells than a protein-based vaccine, and may express a trimeric HA antigen in the membrane of the transduced cell that may have a more native structure than the HA in MIV. The duration and location of antigen presentation may also differ from MIV. These features may lead to a B-cell repertoire with different specificities and a broader expansion of antigen-specific B cells that could produce higher magnitude responses with higher avidity.^23^ In this trial, compared to subjects who received only H7N9 MIV twice, subjects receiving H7 DNA as a priming vaccine developed higher HAI and neutralizing antibody titers 2 weeks following the H7N9 MIV boost. While this trial contained a limited number of subjects, these results are consistent with prior studies evaluating a similar regimen for H5N1, which showed that H5 DNA priming for an H5N1 MIV vaccine improved the antibody response when the boost interval was greater than 12 weeks.^22, 24^

An HAI titer of ≥1:40 is the traditional benchmark used as a correlate for protection against seasonal influenza and as one criterion for licensing seasonal influenza vaccines.^12^ However, it remains unclear what level of HAI titer is associated with protection against novel influenza strains including H7N9. Serologic evaluation of laboratory-confirmed cases of H7N9 in China suggest that robust antibody responses are associated with increased survival. In one cohort of 45 patients infected with H7N9, 64.9% of survivors had H7N9 HAI titers ≥80 while among fatal cases, only 28.6% had HAI titers ≥80 (ref. 31). In contrast, an additional serological evaluation of 18 patients with confirmed H7N9 infection revealed that early and rapid induction of neutralizing antibodies, rather than HAI, correlated with improved clinical outcome.^32^ In our study, 50% of those receiving the heterologous vaccine regimen of H7 DNA prime-H7N9 MIV boost achieved HAI titers ≥1:40 following the boost compared with only 1 of 9 receiving H7N9 MIV prime alone and boost. The HAI titers of the subjects were compared against both the Anhui and Shanghai strains. In subjects that received the H7 DNA and H7N9 MIV prime concurrently, there was a significant increase compared to the H7N9 MIV prime alone group for the Shanghui strain (14.7 vs. 5.8, *p* = 0.03) and marginal significance for the Anhui strain (21.6 vs. 8.3, *p* = 0.063). Nevertheless, the trends are very similar over the two strains. This difference in significance may have occurred due to the small number of subjects involved in the study.

Previous studies have indicated that older individuals may suffer from immunosenescence following vaccination.^33^ While our study did contain a small number of older subjects, upon enrollment they were block randomized stratified by age to prevent any bias. Upon analysis, the HAI titers for these older subjects were found to be comparable to those of the younger subjects; suggesting that there was no age effect on the vaccine response in this study.

In addition, the magnitude of vaccine induced neutralizing antibody responses was 4 to 5-fold greater in the subjects who received the DNA prime. The immunogenicity of the H7 DNA prime was not negatively impacted by co-administration of the H7N9 MIV prime. Subjects who received both the H7 DNA and H7N9 MIV prime concurrently achieved higher neutralizing antibody titers prior to the boost compared with those receiving only the H7 DNA prime, suggesting that the combination priming regimen may provide earlier protection (Fig. 3).

Since the emergence of H7N9 in China in 2013, several candidate vaccines have been evaluated in humans including a MF59-adjuvanted, cell-culture-derived H7N9 monovalent subunit vaccine that induced HAI titers ≥1:40 in 52% of recipients following a 2-dose strategy,^34^ an ISCOMATRIX-adjuvanted, recombinant virus-like particle Influenza A/H7N9 vaccine that achieved HAI titers ≥1:40 in up to 80.6% of those receiving two doses,^35^ and an AS03-adjuvanted monovalent inactivated H7N9 vaccine that elicited HAI titers ≥1:40 in up to 84% of recipients following a two-dose regimen.^36^ These vaccines, however, required multiple doses as well as an adjuvant to achieve immunogenicity. While the vaccine strategy described here still requires more than one dose, this study did not require adjuvants and included a DNA vaccine platform. The advantages of DNA vaccine platforms include: lack of anti-vector immunity, plasmid DNA stability, speed and ease of manufacturing and enhanced antibody and T-cell responses when administered via needle-free delivery system.^37, 38^ Previous experience with DNA vaccines shows them to be safe, well-tolerated and immunogenic as a priming vaccine, and they may be optimally suited for pre-pandemic scenarios where pre-existing immunity is lacking in the population.^22, 28, 38–40^

In conclusion, the administration of H7 DNA and H7N9 MIV vaccines in prime-boost regimens was safe and well tolerated. Subjects that received H7 DNA as a priming vaccine developed higher HAI and neutralizing antibody titers 2 weeks following the H7N9 MIV boost. While the small trial size is a limitation to this study, the results do agree with previous studies involving DNA priming vaccines.^22, 24^ Although demonstrated in multiple clinical trials, the mechanisms underlying this DNA priming effect remain poorly understood and continue to warrant evaluation. Since the population is generally naive to H7, this provided the unique opportunity to evaluate immune responses to novel influenza vaccine platforms in the absence of baseline immunity. Further evaluation of the DNA prime-inactivated protein boost regimen on the diversity of the B cell repertoire, and the ability to elicit stem-specific antibodies capable of neutralizing heterologous influenza virus strains will provide additional insight into the complex immunological response to influenza vaccination.

## Methods

### Study design and participants

VRC 315 was a single-site, phase 1, open label, randomized clinical trial performed at the National Institutes of Health (NIH) Clinical Center by the National Institute of Allergy and Infectious Diseases (NIAID) Vaccine Research Center (VRC), NIH, Bethesda, MD, USA. The study was designed to assess the recombinant H7 DNA plasmid vaccine, VRC-FLUDNA071-00-VP, administered alone or with the monovalent influenza subunit virion H7N9 Vaccine (MIV) as prime with H7N9 MIV boost compared to H7N9 MIV prime with H7N9 MIV boost. VRC 315 was designed to examine the safety, tolerability, and immunogenicity of prime-boost vaccination regimens against H7N9 influenza, in healthy adults aged 18–60 years with no previous H7 avian influenza investigational vaccine administration. Subject inclusion criteria also included a body-mass index lower than 40, normal baseline blood counts, and normal liver and renal function laboratory measurements. The study was reviewed and approved by the NIAID Institutional Review Board. Individuals provided written informed consent and completed a study specific assessment of understanding before enrollment. Human subjects protection guidelines for conducting clinical research from the US Department of Health and Human Services were followed. The primary end points evaluated the safety and tolerability of the three prime-boost regimens. The primary immunogenicity time point was 2 weeks after the H7N9 MIV boost. Sample size was determined to ensure good precision in estimating severe adverse event rate. Secondary and exploratory end points evaluated H7-specific antibody responses assessed by HAI assay and pseudovirus neutralization assays.

### Randomization and masking

During enrollment, study participants were randomly assigned as per protocol design (Fig. 1) with a computer generated block randomization stratified by age. The study statistician and pharmacists developed and maintained the randomization code.

### Vaccines

The H7 DNA vaccine (VRC-FLUDNA071-00-VP) was manufactured for the VRC by Leidos Biomedical Research, Inc., Frederick, MD at the VRC/NIAID Pilot Plant and consists of a single, closed-circular plasmid DNA macromolecule (VRC-3601), that encodes the hemagglutinin 7 (H7) protein of A/Anhui/1/2013 (H7N9) influenza derived from a human isolate and identified as EpiFlu Accession # EPI439507 in the Global Initiative on Sharing All Influenza Data (GISAID) EpiFlu database. The plasmid was synthesized by GeneScript (Piscataway, NJ) using human preferred codons as previously described^41, 42^ and contains a CMV/R promoter as previously described.^43^ The plasmid DNA was prepared under current Good Manufacturing Practices at 4 mg/mL in phosphate buffered saline.

Monovalent influenza subunit virion (MIV) A/Shanghai/2/2013 (H7N9) influenza vaccine was produced by and sourced from Sanofi Pasteur, Inc. (Swiftwater, PA). The vaccine reference reassortant A/Shanghai/2/2013 used for vaccine production was provided by the Centers for Disease Control and Prevention (CDC), and the production was completed following the procedures and methods used to manufacture licensed influenza virus vaccines such as Fluzone® (Sanofi Pasteur, Inc.). The HA of Shanghai is extremely similar to Anhui, with only two nucleotide differences that result in a single amino-acid change. Monovalent bulk concentrate production, bulk formulation of the vaccine, and filling operations were all performed in the licensed facilities. The vaccine was produced in eggs; antibiotics were not used in the manufacturing of the vaccine, and it contains no preservative, no latex or adjuvant.

### Procedures

Thirteen males and 17 females were enrolled in VRC 315, into one of three groups to determine the safety, tolerability, and immunogenicity of prime-boost vaccination regimens against H7N9 Influenza. Group 1 received H7 DNA at day 0 and H7N9 MIV at week 16. Group 2 received H7 DNA and H7N9 MIV at day 0 followed by H7N9 MIV at week 16. Group 3 received H7N9 MIV at day 0 and week 16 (Fig. 1). All DNA vaccinations were 4 mg and given via a pressurized needle-free delivery system (Biojector^®^ 2000 Needle-Free Injection Management System) and all H7N9 MIV vaccinations were 45 μg, administered by needle and syringe. Group 2 participants received the H7 DNA and H7N9 MIV as two injections administered in different arms on Day 0. The dosages of DNA vaccine and MIV vaccine were based on previous trials^22, 28^ as well as previous human experience with inactivated H7 vaccines.^13, 15^ All injections were given intramuscularly in the deltoid. We assessed local and systemic reactogenicity for 7 days after each vaccination. We recorded all adverse events for 28 days after each vaccination and coded the adverse events using the Medical Dictionary for Regulatory Activities (severity scale 0–5). Outside of these 28-day windows, only serious adverse events, new chronic medical conditions, and influenza or influenza-like illness were recorded.

Sera samples were collected at 0, 4, 16, 17, 18, 20, and 28 weeks. Subject sera were evaluated to determine the relative concentration of A/Anhui/1/2013 (H7N9) neutralizing antibodies by evaluation of the capacity of subject serum to prevent the infection of 293 A cells by replication incompetent HA-pseudotyped virus (A/Anhui/1/2013 H7) as previously described.^22^ In all, 293 A cells were procured from American Type Culture Collection, from which master and working cell banks were constructed and utilized for testing. Cells were tested monthly for Mycoplasma contamination. Neutralization activity was quantitated by relative decrease in the luciferase activity as compared to infection of cells in the absence of subject sera and the 80% inhibition serum titer (ID_80_) was calculated relative to the signal in the absence of sera using five-parameter curve fitting.

The HAI assays were done in V-bottom 96-well plates using four hemagglutinating units of virus and 1% horse red blood cells following the protocol established by WHO Collaborating Center for Reference and Research on Influenza at the Centers for Disease Control and Prevention, USA (https://consise.tghn.org/articles/consise-and-avian-influenza-h7n9/). The PR8 reassortant viruses with HA and NA from A/Anhui/1/2013 or A/Shanghai/2/2013, kindly provided by Li-Mei Chen at the Centers for Disease Control and Prevention, Influenza Division were used in HAI assay under BSL2 + laboratory conditions.

Methods were performed in accordance with relevant guidelines and regulations.

### Statistical analysis

The primary outcomes of the study related to safety, tolerability, and immunogenicity of the three prime-boost vaccination regimens. We reported positive response rates along with exact 95% confidence intervals computed by the Pearson-Clopper method. Antibody response data were close to being log normally distributed. We reported the magnitude of antibody response via geometric mean and 95% confidence interval. Group comparisons with respect to the magnitude of response were based on one-way ANOVA and two-sided two-sample *t*-test on log transformed data. Group comparisons with respect to the positive response rate were based on Fisher’s exact test. Statistical computations were done by statistical software SAS and R.

### Availability of data

The underlying data reported in this paper is available through the corresponding author.

## Electronic supplementary material

Supplementary Clinical Trial Protocol

Supplementary Figure S1, Table S1, Table S2
