# Supplementary material for: An avian influenza H7 DNA priming vaccine is safe and immunogenic in a randomized phase I clinical trial
Source: NPJ Vaccines. 2017 Jun 1;2:15. doi: 10.1038/s41541-017-0016-6 (PMC5627236; doi:10.1038/s41541-017-0016-6)
Supplement: Supplementary file 1 — Supplementary Clinical Trial Protocol [file 41541_2017_16_MOESM1_ESM.pdf]

Version 3.0
February 6, 2015

VACCINE RESEARCH CENTER

Protocol VRC 315
(NIH 14-I-0160)

A Phase I Open-Label, Randomized Study of H7 Influenza Prime-Boost Regimens in Healthy Adults: Recombinant H7 DNA Plasmid Vaccine, VRC-FLUDNA071-00-VP, Administered Alone or with Monovalent Influenza Subunit Virion H7N9 Vaccine (MIV) as Prime with MIV Boost Compared to MIV Prime with MIV Boost

Vaccines Provided by
National Institute of Allergy and Infectious Diseases (NIAID)
Vaccine Research Center (VRC)
Bethesda, Maryland
and
Sanofi Pasteur, Inc.
Swiftwater, Pennsylvania

Clinical Trial Sponsored by:
National Institute of Allergy and Infectious Diseases (NIAID)
Vaccine Research Center (VRC)
Bethesda, Maryland

BB-IND 16053– held by VRC, NIAID, NIH

Principal Investigator:
Julie Ledgerwood, D.O.
Vaccine Research Center,
National Institute of Allergy and Infectious Diseases (NIAID)
National Institutes of Health (NIH)
Bethesda, MD 20892

IRB Initial Review Date: June 30, 2014

TABLE OF CONTENTS

Page
Table of Abbreviations 5
Précis 7
1. INTRODUCTION AND RATIONALE **8**
1.1 Influenza Biology and Natural History 8
1.2 Influenza Vaccines: Current and Developing Technologies 9
1.3 Rationale for Development of Avian H7 Influenza Plasmid DNA Vaccine 9
1.4 Rationale for Avian H7 Influenza Plasmid DNA Vaccine Prime-Inactivated H7N9 MIV Boost 9
1.5 Previous Human Experience with VRC DNA vaccines 10
1.5.1 Plasmid DNA Vaccines Developed by VRC, NIAID, NIH 10
1.5.2 Influenza DNA Vaccines Developed by VRC, NIAID, NIH 10
1.6 Previous Human Experience with Inactivated H7 Vaccines 12
1.7 Assessment of Immunogenicity 13
2. BACKGROUND ON VACCINES 13
2.1 Formulation, Manufacture, and Packaging of VRC-FLUDNA071-00-VP 13
2.2 Formulation, Manufacture, and Packaging of Inactivated H7N9 MIV 14
2.3 Summary of preclinical immunogenicity study of VRC-FLUDNA071-00-VP in Mice.. 14
2.4 Preclinical Studies with H7N9 MIV 14
3. STUDY OBJECTIVES 14
3.1 Primary Objective 15
3.2 Secondary Objectives 15
3.3 Exploratory Objectives 15
4. STUDY DESIGN AND METHODS 15
4.1 Study Population 16
4.1.1 Inclusion Criteria 16
4.1.2 Exclusion Criteria 17
4.2 Subject Monitoring: Schedules of Evaluations 18
4.2.1 Screening 19
4.2.2 Study Schedule and Randomization 19
4.2.3 Administration of Injections 19
4.2.4 Diary Cards and Follow-up 20
4.2.5 Blood Sample Collection 20
4.2.6 Concomitant Medications 21
4.3 Criteria for Discontinuing Study Injections or Protocol Participation 21
4.3.1 Discontinuation of Study Injections 21
4.3.2 Discontinuation from Protocol Participation 22
4.4 Criteria for Pausing the Study 22
5. SAFETY AND ADVERSE EVENT REPORTING 23
5.1 Adverse Events 23

5.2 Serious Adverse Events 23
5.3 Adverse Event Reporting to the IND Sponsor 24
5.3.1 IND Sponsor Reporting to the FDA 24
5.4 Reporting to the Institutional Review Board 24
5.4.1 Unanticipated Problem (UP) Definition 24
5.4.2 Protocol Deviation Definition 25
5.4.3 Non-Compliance Definition 25
5.4.4 Expedited Reporting to the NIAID IRB 25
5.4.5 Annual Reporting to the NIAID IRB 26
5.5 Serious Adverse Event Reporting to the Institutional Biosafety Committee 26

6. STATISTICAL CONSIDERATIONS 26

6.1 Overview 26
6.2 Objectives 26
6.3 Endpoints 26
6.3.1 Primary Endpoints: Safety 26
6.3.2 Secondary Endpoints: Immunogenicity 27
6.3.3 Exploratory Endpoints 27
6.4 Sample Size and Accrual 27
6.4.1 Power Calculations for Safety 27
6.4.2 Sample Size Calculations for Immunogenicity 28
6.4.3 Power for Comparison 29
6.5 Statistical Analysis 29
6.5.1 Analysis Variables 29
6.5.2 Baseline Demographics 29
6.5.3 Safety Analysis 29
6.5.4 Immunogenicity Analysis 30
6.5.5 Interim analyses 30
6.5.6 Randomization of Treatment Assignments 31

7. PHARMACY AND VACCINE ADMINISTRATION PROCEDURES 31

7.1 Study Agents 31
7.2 Study Agent Presentation and Storage 31
7.2.1 Study Agent Labels 31
7.2.2 Study Agent Storage: 32
7.3 Preparation of Study Agent for Injection 32
7.4 Study Agent Accountability 33
7.4.1 Documentation 33
7.4.2 Disposition 33

8. HUMAN SUBJECT PROTECTIONS AND ETHICAL OBLIGATIONS 33

8.1 Institutional Review Board 33
8.2 Subject Recruitment and Enrollment 33
8.3 Informed Consent 34
8.4 Subject Confidentiality 34
8.5 Risks and Benefits 34
8.5.1 Risks of the Monovalent Inactivated H7N9 Vaccine 34

8.5.2 Risks of the H7 DNA vaccine ... *35*
8.5.3 Other Risks ... *35*
8.5.4 Study Benefits ... *36*
8.6 Plan for Use and Storage of Biological Samples ... 36
8.6.1 Use of Samples, Specimens and Data ... *36*
8.6.2 Storage and Tracking of Blood Samples and Other Specimens ... *36*
8.6.3 Disposition of Samples, Specimens and Data at Completion of the Protocol ... *37*
8.6.4 Loss or Destruction of Samples, Specimens or Data ... *37*
8.7 Subject Identification and Enrollment of Study Participants ... 37
8.7.1 Participation of Children ... *37*
8.7.2 Participation of NIH Employees ... *37*
8.8 Compensation ... 38
8.9 Safety Monitoring, Protocol Safety Review Team ... 38

9. ADMINISTRATIVE AND LEGAL OBLIGATIONS ... 38

9.1 Protocol Amendments and Study Termination ... 38
9.2 Study Documentation and Storage ... 38
9.3 Data Collection and Protocol Monitoring ... 39
9.3.1 Data Collection ... *39*
9.3.2 Source Documents ... *39*
9.3.3 Protocol Monitoring Plan ... *39*
9.4 Language ... 40
9.5 Policy Regarding Research-Related Injuries ... 40

10. REFERENCES ... 41

APPENDICES

I Informed Consent Form ... 44
II Contact Information ... 54
III Schedules of Evaluations ... 56
IV Table for Grading Severity of Adverse Events ... 58

ABBREVIATIONS

Abbreviation	Term
AE	adverse event
ALT	alanine aminotransferase
AoU	assessment of understanding
Biojector	Biojector® 2000 Needle-Free Injection Management System
BMI	body mass index
CBC	complete blood count
cGMP	current Good Manufacturing Practices
CMV	Cytomegalovirus
CMV-IE	cytomegalovirus early region 1 enhancer
CMV/R	CMV promoter substituted with a portion of the HTLV-1 long terminal repeat (LTR)
DNA	deoxyribonucleic acid
ELISA	enzyme-linked immunosorbent assay
ELISPOT	enzyme-linked immunospot
FDA	Food and Drug Administration
GCP	Good Clinical Practices
GLP	Good Laboratory Practices
GMT	Geometric Mean Titer
H7	hemagglutinin 7
H7N9	hemagglutinin 7, neuraminidase 9
HA	Influenza hemagglutinin protein
HAI	hemagglutinin inhibition
HIV	human immunodeficiency virus
HLA	human leukocyte antigen
HTLV-1	human T cell leukemia virus type 1
IB	Investigator’s Brochure
IBC	Institutional Biosafety Committee
ICS	intracellular cytokine staining
IM	Intramuscular
IND	investigational new drug application
IRB	Institutional Review Board
LIMS	Laboratory Information Management System
LTR	long terminal repeat
MedDRA	Medical Dictionary for Regulatory Activities
MIV	Monovalent Inactivated Vaccine
NA	influenza neuraminidase protein
NIAID	National Institute of Allergy and Infectious Diseases
NIH	National Institutes of Health
NSAID	nonsteroidal anti-inflammatory drug
NVITAL	NIAID Vaccine Immune T-Cell and Antibody Laboratory
PBMC	peripheral blood mononuclear cells
PBS	phosphate buffered saline

PI	Principal Investigator
PREP	Public Readiness and Emergency Preparedness
RNA	ribonucleic acid
RVP	reporter virus particle
SAE	serious adverse event
SARS	severe acute respiratory syndrome
SAS	Statistical Analysis System
SUSAR	serious and unexpected suspected adverse reaction
TIV	trivalent inactivated vaccine (for seasonal influenza)
ULN	upper limit of normal
UP	Unanticipated Problem
VRC	Vaccine Research Center
VPP	VRC/NIAID/Vaccine Pilot Plant
WBC	white blood cell
WHO	World Health Organization
WNV	West Nile virus

Précis

VRC 315 Study: A Phase I Open-Label, Randomized Study of H7 Influenza Prime-Boost Regimens in Healthy Adults: Recombinant H7 DNA Plasmid Vaccine, VRC-FLUDNA071-00-VP, Administered Alone or with Monovalent Influenza Subunit Virion H7N9 Vaccine (MIV) as Prime with MIV Boost Compared to MIV Prime with MIV Boost

Study Design: This is a Phase I, randomized, open-label study to evaluate the safety, tolerability, and immunogenicity of prime-boost vaccination regimens against H7N9 Influenza. A vaccination regimen with the VRC-FLUDNA071-00-VP (H7 DNA) vaccine administered alone or concurrently in different arms with A/Shanghai/2/2013(H7N9) (MIV) as prime followed by inactivated MIV boost will be compared to MIV-MIV prime-boost with a 16 week boost interval. The primary hypothesis is that all the H7 DNA prime-MIV boost, DNA+MIV prime-MIV boost, and MIV-MIV study regimens will be safe for human administration. A secondary hypothesis is that the H7 DNA-MIV regimen will elicit a greater frequency and/or magnitude of antibody against H7 than the MIV-MIV regimen. The primary objective is to evaluate the safety and tolerability in healthy adults of the investigational vaccine regimens. Secondary objectives are related to the immunogenicity of the vaccination regimens.

Product Description: The investigational VRC-FLUDNA071-00-VP vaccine was developed by VRC, NIAID and is composed of a single closed-circular DNA plasmid that encodes the H7 hemagglutinin (HA) protein of A/Anhui/1/2013 (H7N9) influenza, and is supplied in single dose vials at a concentration of 4 mg/mL. H7 DNA vaccinations will be 4 mg administered as a 1 mL intramuscularly (IM) using the Biojector® 2000 Needle-Free Injection Management System (Biojector). The inactivated H7N9 vaccine is monovalent subunit virion vaccine, A/Shanghai/2/2013(H7N9) MIV, manufactured by Sanofi Pasteur, Inc (Swiftwater, PA), and supplied at 30 mcg/0.5 mL per vial. The H7N9 MIV vaccinations will be 45 mcg administered as a 0.75 mL IM injection using needle and syringe. All injections will be in the deltoid muscle.

Subjects: A total of up to 36 healthy adults, ages 18-60 years will be enrolled.

Study Plan: Subjects will be randomized equally into the three study groups and will receive two vaccinations as shown in the schema. The protocol requires 8 clinic visits and a telephone follow-up contact after each study injection. Durability of immune response will be followed through Study Week 28.

**VRC 315 Study Schedule**			
**Group**	**Subjects***	**Day 0**	**Week 16 (±4 weeks)**
Group 1	10	H7 DNA	H7N9 MIV
Group 2	10	H7 DNA+H7N9 MIV	H7N9 MIV
Group 3	10	H7N9 MIV	H7N9 MIV
Total	30	H7 DNA injections are 4 mg administered as 1 mL IM by Biojector. H7N9 MIV injections are 45 mcg administered as 0.75 mL IM by needle/syringe. Prime injections in Group 2 will be administered in different arms.	
*Up to 6 subjects over target enrollment are permitted in the study. Randomizations will be stratified by age; up to 9 subjects ages 51-60 years will be enrolled.			

Study Duration: Subjects will be evaluated for safety and immune responses throughout the study for 12 weeks following the boost. Duration of time on study will be 28 weeks.

1. INTRODUCTION AND RATIONALE

1.1 INFLUENZA BIOLOGY AND NATURAL HISTORY

Influenza is a negative-strand ribonucleic acid (RNA) virus that belongs to the family *Orthomyxoviridae*. Of the three genera of influenza circulating in nature (influenza A, B, and C), only the first two are known to cause epidemics [1]. Influenza A viruses are encoded by 8 RNA gene segments, and classified on the basis of the antigenicity of their surface glycoproteins: hemagglutinin (HA) and neuraminidase (NA). There are 18 different HA subtypes and 11 NA subtypes known to exist, but only three HA subtypes (H1, H2, and H3) and two NA subtypes (N1 and N2) have caused significant human epidemics [2]. Emerging novel avian influenza strains such as H5N1 and H7N9 have also caused severe infections in humans and prompt serious public health concerns.

The public health burden of influenza in the world is enormous. Annual influenza epidemics cause about 250,000 to 500,000 deaths worldwide [3]. Circulating viruses change quickly and re-assort with each other creating new viruses. These present an immediate threat to public health and currently require the preparation of new vaccines directed at the changing viral strains that are prevalent annually. Emerging virus strains present the potential of a pandemic when there is little or no pre-existing immunity in the population as observed in worldwide outbreaks of influenza in the last century [4].

The first notification of human infection with H7N9 avian influenza came from China at the end of March 2013. As of February 28, 2014, 375 laboratory confirmed cases of human infection with avian influenza A(H7N9) including 115 deaths have been reported to World Health Organization (WHO) from 13 provinces in eastern mainland China and Hong Kong, and one case in a Chinese traveler was reported from Malaysia [5]. Most of the cases were severe respiratory illnesses with about 30% resulting in death [5, 6]. The infections have been reported in both men and women across a wide age range, with most cases occurring in middle-aged and older men, and few in children, teenagers and young adults [5]. As per CDC, no evidence of sustained person-to-person spread of H7N9 has been found, although some evidence points to limited person-to-person spread in rare circumstances [6]. The emerging H7N9 virus has not been detected in people or birds in the United States [6], however, H7N9 type viruses have been isolated from domestic poultry on several occasions [7]. It has been suggested that the emerging H7N9 virus occurred as a result of multiple re-assortments in wild birds and domestic poultry.

There are many unknowns about this novel emerging H7N9 influenza strain, and intensive epidemiological and virological surveillance is ongoing to elucidate the animal reservoirs and routes of transmission to humans [7, 8]. The current evidence suggests that transmitted viruses remain antigenically similar to the A/Anhui/1/2013 (H7N9) prototype [5]. Although avian influenza A(H7N9) viruses do not transmit easily from animals to humans, their transmissibility may be greater than the transmissibility of avian influenza A(H5N1) viruses as judged by frequency of detection in the poultry markets that were considered to be the source of the initial outbreak in 2013 compared to the number of human cases [5].

The reported morbidity and mortality of H7N9 infection, and the potential for human-to-human transmission is an incentive to proceed with development of a human vaccine for H7N9 avian influenza.

1.2 Influenza Vaccines: Current and Developing Technologies

Currently, licensed seasonal influenza vaccines consist of 3 components: an H1N1, H3N2, and one or two influenza B virus strains. Beginning with the 2013-14 vaccines, quadrivalent influenza vaccines containing two influenza B virus strains were licensed for use in the U.S. These vaccines depend upon labor-intensive methods and limited manufacturing capacity, and often induce suboptimal immunity in vulnerable populations of children, older adults and immunocompromised individuals [9-11].

There is serious concern that the currently available production methodology cannot meet world-wide public health needs to produce sufficient quantities of highly immunogenic avian influenza vaccine if it were needed. Also, influenza antigen evolution may necessitate a more agile, rapidly scalable production process. In an effort to overcome the limitations of egg-based influenza vaccine production methods, manufacturers are developing cell culture systems. Two novel influenza vaccines made with alternative manufacturing technologies, Flucelvax (Novartis, via mammalian cell line, approved in November 2012, [12]) and Flublock (Protein Sciences Corporation, via recombinant DNA technology to create a recombinant baculovirus-expressed HA, approved in January 2013, [13]) are now licensed in the U.S.

There is a limited experience with vaccines targeted to H7 in humans, with data available for two phase 1 clinical studies so far (Section 1.6). Both of these studies indicated that inactivated vaccines that incorporate H7 are safe and well tolerated in humans but induced suboptimal immune responses. This is similar to the data generated in prior H5N1 vaccine trials, further confirming that immune responses to vaccines against novel avian influenza strains are generally lower than those seen against most seasonal influenza vaccines.

Several new technologies are being currently tested in preclinical animal studies such as a synthetic consensus H7 DNA vaccine [14], a recombinant Newcastle disease virus expressing H7 [15], and a virus-like particle H7N9 vaccine [16].

1.3 Rationale for Development of Avian H7 Influenza Plasmid DNA Vaccine

DNA vaccines have the potential to be manufactured rapidly. They are known to induce balanced immune responses that induce both humoral and cellular immunity. The potential and experience to date with DNA vaccines warrants continued investigation of DNA vaccines as safe and efficient technology for responding to emerging infectious diseases [17].

Investigators at the Vaccine Research Center (VRC), National Institute of Allergy and Infectious Diseases (NIAID), National Institutes of Health (NIH), Bethesda, MD have developed a plasmid DNA vaccine strategy that has demonstrated protective immune responses against influenza in preclinical studies [18, 19]. This vaccine platform was also tested for safety and immunogenicity in human clinical trials, and provides the technology platform for development of additional influenza vaccine products used as part of a prime-boost regimen [20-22].

VRC-FLUDNA071-00-VP is a plasmid DNA vaccine developed by VRC and intended for use as a preventive vaccine for avian H7N9 influenza virus infection.

1.4 Rationale for Avian H7 Influenza Plasmid DNA Vaccine Prime-Inactivated H7N9 MIV Boost

In this study we will evaluate a prime-boost approach based on data generated by the VRC in two human trials assessing a candidate DNA H5 vaccine. H7 DNA prime-H7N9 MIV boost

with a 16 week interval will be evaluated and compared to MIV prime-MIV boost with the same prime-boost interval. In previous studies using a boost interval of 12-24 weeks for a H5 DNA prime—H5N1 MIV boost regimen, the VRC has found that antibody responses were 4-6 fold higher compared to MIV-MIV regimens [21-23]. Based on this work, a H7 DNA prime followed by a H7N9 MIV boost with a 16 week interval is being evaluated in VRC 315 towards the goal of an influenza vaccination strategy with improved immunogenicity.

It is important to achieve early protection in a pandemic situation, however, our experience with HA DNA vaccines is that we do not see impressive immune responses before the boost. In animal studies, rabbits were immunized by HA DNA vaccine, licensed TIV, or combination of both followed by TIV administered as a boost (DNA-TIV) or TIV co-administered with HA DNA (DNA+TIV-DNA+TIV) as a boost in 4 weeks. The results indicated that there was no boosting effect upon co-administration of DNA and TIV, but the responses after two immunizations were comparable in DNA-TIV and DNA+TIV-DNA+TIV groups [24]. To investigate a temporal pattern for immune response development, Group 2 subjects will have an H7 DNA vaccine and MIV administered in different arms on Day 0 followed by a boost with MIV at week 16.

The investigational H7 DNA vaccine in this study encodes for H7 of the A/Anhui/1/2013 (H7N9) influenza strain, and the MIV for the boost is the investigational Sanofi Pasteur product based on A/Shanghai/2/2013(H7N9) strain. These H7N9 influenza strains are very close in sequence, and therefore it is expected that A/Shanghai/2/2013(H7N9) MIV will provide an efficient boost for H7 DNA vaccine based on the A/Anhui/1/2013 (H7N9) sequence [25].

1.5 PREVIOUS HUMAN EXPERIENCE WITH VRC DNA VACCINES

1.5.1 Plasmid DNA Vaccines Developed by VRC, NIAID, NIH

Investigators at the VRC/NIAID/NIH in Bethesda, MD have evaluated plasmid DNA vaccine strategies since 2001 in preclinical and clinical studies. Dosages up to 8 mg have been administered, with the majority of injections being at a 4 mg dosage, which offers the combination of a good safety profile, greater ease of administration than an 8 mg dosage, and optimal immunogenicity as indicated by immune response. The results of human clinical trials with VRC DNA vaccines have been published for HIV DNA vaccines [26-31], an Ebola virus DNA vaccine [32], a SARS DNA vaccine [33], West Nile virus DNA vaccines [34, 35] and H5 DNA vaccine [21, 22]. VRC clinical trials of WNV, SARS and H5 DNA vaccines have provided evidence that a DNA vaccine can induce neutralizing antibody as assessed by a reporter virus particle (RVP) neutralizing antibody assay [21, 22, 33-35]. Biojector® 2000 needle-free administration improves the immune response for DNA vaccine delivery compared to using a standard needle and syringe [36]. IM and SC delivery of vaccines via Biojector is cleared for use by the FDA in adults and children.

1.5.2 Influenza DNA Vaccines Developed by VRC, NIAID, NIH

The VRC, NIAID, NIH has developed several investigational influenza DNA vaccine products. Clinical evaluation of the first influenza DNA product was initiated in 2006 with an avian influenza H5 DNA vaccine. Through a series of clinical trials, influenza DNA vaccines have been tested alone or in prime-boost regimens with the respective inactivated vaccines in the clinical trials as shown in **Table 1**:

Table 1: Experience with VRC Influenza DNA Vaccines in Adult and Children Populations

IND	IND Sponsor	Product	Subjects, N (years)	Boost / Interval	VRC study
BB-IND 13197	RCHSPB/ NIAID	VRC-AVIDNA036-00-VP	45 (18-60)	N/A	**VRC 304**
			44 (18-60)	N/A	**VRC 305**
BB-IND 13836	RCHSPB/ NIAID	VRC-AVIDNA036-00-VP	60 (18-60)	MIV (4, 24 weeks)	**VRC 306**
			64 (18-60)	MIV (4-24 weeks)	**VRC 310**
BB-IND 13939	*VRC/NIAID	VRC-FLUDNA047-00-VP	51 (18-70)	TIV (4 weeks)	**VRC 307**
		VRC-FLUDNA056-00-VP	60 (45-70)	TIV (3 weeks)	**VRC 309**
		VRC-FLUDNA061-00-VP	131 (18-70)	^TIV (36 weeks)	**VRC 701**
BB-IND 14093	RCHSPB/ NIAID	VRC-FLUDNA057-00-VP	20 (18-70)	MIV (3-17 weeks)	**VRC 308**
BB-IND 15062	VRC/NIAID	VRC-FLUDNA063-00-VP	75 (6-17)	TIV (18 weeks)	**VRC 702**
BB-IND 15147	VRC/NIAID	VRC-FLUDNA063-00-VP	316 (18-70)	TIV (14 weeks)	**VRC 703**
BB-IND(TBD)	VRC/NIAID	VRC-FLUDNA071-00-VP	24 (18-60**)**	MIV (16 weeks)	**VRC 315**

*IND initiated with RCHSPB/NIAID Sponsorship; transferred to VRC/NIAID in 2011.
^TIV for the next influenza season mismatched in 2 strains
More information on these studies can be found in the Investigator's Brochure (IB).

<u>H5 DNA Vaccine in prime-boost regimens (BB-IND 13836)</u> was evaluated in two Phase 1 studies (VRC 306 and VRC 310) in healthy adults ages 18-60 years old. Between these two studies the prime-boost regimens evaluated included two doses of monovalent inactivated vaccine (MIV, Sanofi Pasteur H5N1 A/Indonesia 05/05 avian influenza vaccine) at 4 or 24 week intervals or H5 DNA vaccine followed by MIV at varying intervals from 4 to 24 weeks. Cumulatively, 124 subjects were enrolled. Between the two studies, 114/114 (100%) of the expected H5 DNA injections were administered to 99 subjects randomized to schedules with H5 DNA primes and 147/149 (99%) of the expected H5N1 MIV injections were administered to 122 subjects.

There was no severe local or systemic reactogenicity. Mild local reactogenicity was reported in a majority of the H5 DNA vaccine recipients (83%) while 2% reported moderate local reactogenicity. The majority of the H5 DNA vaccine recipients reported no systemic reactogenicity; 23% reported mild and 5% reported moderate systemic reactogenicity. No SAE were reported following the H5 DNA vaccinations; there was one SAE reported in the follow-up period through 48 weeks: multiple injuries unrelated to study participation in the period after the H5N1 booster. All adverse events following H5 DNA injection were mild (Grade 1) or moderate (Grade 2) except for one severe (Grade 3) case of gastroenteritis with onset 15 days after H5 DNA vaccination, which was assessed as unrelated to vaccine.

In assessing immunogenicity of H5 DNA vaccine followed by MIV, a single H5 DNA vaccine prime followed by a single MIV boost at short intervals (4-8 weeks) did not significantly improve HAI titers over that achieved with inactivated vaccine alone. However, a single H5 DNA 4 mg vaccination prime significantly improved HAI responses when the interval to the inactivated vaccine boost was 12-24 weeks, as compared with two vaccinations with the inactivated vaccine [21, 22]. Therefore, a single dose of DNA priming may be sufficient to increase the magnitude and breadth of HA-specific antibody responses.

Summary of HA DNA safety and immunogenicity: The safety data for the VRC HA DNA vaccine trials to date shows that these vaccines were well tolerated. No severe local or systemic reactogenicity occurred nor were there any serious adverse events related to vaccine. The pattern of local and systemic reactogenicity appears to be generally mild and similar across the studies, independent of the antigens encoded by the HA DNA constructs included in any one vaccine.

Data from studies of the H5 DNA vaccine using various H5N1 inactivated vaccine (MIV) boost intervals show that DNA-MIV schedules are associated with a higher magnitude of immune response than MIV-MIV schedule when there is a longer interval (12 weeks or greater) between prime and boost [21, 22]. Data from HA DNA trials (seasonal or H5) evaluating DNA vaccines without an inactivated vaccine boost or with a short boost interval (3-4 weeks) did not demonstrate this finding, further confirming the importance of the longer boost interval (12-24 weeks).

1.6 PREVIOUS HUMAN EXPERIENCE WITH INACTIVATED H7 VACCINES

The previous experience with H7 inactivated vaccines includes 2 studies, one with H7N7 and one with H7N1 vaccine. In a multicenter study in the U.S. with unadjuvanted subunit H7N7 vaccine produced by Sanofi Pasteur Inc in eggs using seasonal vaccine production methods, 125 healthy adults were randomized to receive 2 doses of vaccine in a dose range from 7.5 mcg to 90 mcg or placebo. Only 8 of 22 recipients had at least a 4–fold increase in HAI after 2 doses of 90 mcg, and none of them achieved a 1:40 titer [37].

In a study conducted in Norway, H7N1 cell-grown inactivated vaccine was tested at 12 and 24 mcg dosages with aluminum hydroxide as adjuvant. None of 13 recipients that received 2 doses of a 24 mcg adjuvanted vaccine developed a 1:40 titer [38].

In preparation to possible H7N9 pandemic, efforts have been made by WHO and CDC to develop seed strains for A(H7N9) vaccines and release these to the vaccine manufacturers [39]. There are several ongoing studies that are targeted to define the vaccine dosage and to determine if a use of adjuvants may help with vaccine immunogenicity and dose-sparing.

A cell-culture derived inactivated A/H7N9 vaccine produced by Novartis was tested in phase 1 study alone at 15 mcg dose or adjuvanted with MF59 at 15, 7.5 and 3.75 mcg dosages [40]. A total of 402 subjects ages 18-64 years were enrolled and all had undetectable HAI titers against A/H7N9 influenza at baseline. The responses to a first dose were very low as expected in the immunologically naïve population, and higher responses were seen with adjuvanted vaccine in comparison to non-adjuvanted. The results indicated that the HAI titers ≥1:40 can be achieved in up to 53% of subjects in groups that received MF59-adjuvanted vaccine, and only 3% of subjects who received non-adjuvanted vaccine have reached the ≥1:40 HAI titer.

In September 2013, two phase 2 studies for evaluation of inactivated H7N9 influenza vaccine were initiated by the Division of Microbiology and Infectious Diseases (DMID), NIAID, NIH at nine clinical sites in the U.S. In these studies, NCT 01938742 and NCT 01942265 at Clinicaltrials.gov, the monovalent A/H7N9 vaccine (A/Shanghai/2/2013) is tested administered alone at dosages 15 mcg and 45 mcg, and at 3.75, 7.5, or 15 mcg with MF 59 or AS03 adjuvants.

A total of 700 and 980 healthy adults ages 19-64 years have been enrolled in DMID 13-0032 study (NCT 01938742) and DMID 13-0033 study (NCT 01942265), respectively. Administration of the second dose of study product has been completed for all eligible study subjects in both studies. Safety follow up of study subjects is ongoing. Overall, the study products were well

tolerated.

Based on the previous human experience, Investigator's Brochure, and experts' recommendation, H7N9 MIV will be used at the 45 mcg dosage in this study.

1.7 ASSESSMENT OF IMMUNOGENICITY

In protocol VRC 315, specimens to evaluate immunogenicity will be collected at baseline and at specified time points. The primary immunogenicity timepoint is 2 weeks after the boost. The HA-specific immune responses will be assessed through 12 weeks after the boost. HA-specific antibody as measured by HAI assay is the traditional benchmark measure of immune response to influenza vaccines and will be conducted on stored samples obtained throughout the study. Additional measurements of antibody and T cell responses may also be assessed from stored samples for timepoints throughout the study as exploratory evaluations.

The detection of antibody by HAI or neutralization assay is based on validated laboratory methods. Exploratory T cell assays may include the ICS assay which is based upon previously published methods [41] and quantitates the frequency of $CD4^+$ and $CD8^+$ cells that produce interleukin-2, interferon-gamma, or TNF-alpha in response to pools of overlapping peptides representing HA antigens. Specific peptides may also be used to detect T cell responsiveness by an ELISpot assay, modified from a previously published method [42].

Research samples for immunogenicity assays will be processed by the NIAID Vaccine Immune T Cell and Antibody Laboratory (NVITAL) in Gaithersburg, MD, where many of the immunogenicity assays will also be performed. Some immunogenicity assays may be performed by VRC laboratories in Bethesda, MD, approved contract laboratories, or approved research collaborators.

2. BACKGROUND ON VACCINES

2.1 FORMULATION, MANUFACTURE, AND PACKAGING OF VRC-FLUDNA071-00-VP

VRC-FLUDNA071-00-VP was manufactured for the VRC by Leidos Biomedical Research, Inc., Frederick, MD (formerly SAIC-Frederick, Inc.) at the VRC/NIAID/Vaccine Pilot Plant (VPP). The VRC-FLUDNA071-00-VP Drug Substance consists of a single closed-circular plasmid DNA macromolecule (VRC-3601), that encodes the hemagglutinin 7 (H7) protein of A/Anhui/1/2013 (H7N9) influenza derived from a human isolate and identified as EpiFlu Accession # EP1439507 in the Global Initiative on Sharing All Influenza Data (GISAID) EpiFlu database.

The plasmid CMV/R promoter consists of translational enhancer region of the CMV immediate early region 1 enhancer (CMV-IE) substituted with the 5′-untranslated human T cell leukemia virus type 1 (HTLV-1) R-U5 region of the human T cell leukemia virus type 1 HTLV-1 long terminal repeat (LTR), and has been shown to optimize gene expression further [43]. This promoter has been evaluated in preclinical safety studies as well as several clinical trials of DNA plasmid vaccines for HIV (BB-IND 11750), WNV (BB-IND 12933), Ebola (BB-IND 11294), SARS (BB-IND 11995) and avian influenza (BB IND 13197).

The plasmid was synthesized by GeneScript (Piscataway, NJ) using human preferred codons as previously described [44]. Viral gene sequences have been modified to optimize expression in human cells.

The process for manufacturing, filling, and packaging VRC-FLUDNA071-00-VP is summarized in the Investigator’s Brochure (IB). Briefly, the plasmid used in the Master Cell Bank (MCB) was synthesized and transferred to the VPP where the sequence was confirmed before use. The plasmid was used to transform the *Escherichia coli* bacterial host strain, DH5α, in order to produce individual MCB. Each MCB was expanded in culture and inoculated into a fermentor for production. Bacterial cell growth was dependent upon the cellular expression of the kanamycin resistance protein encoded by a portion of the plasmid DNA. Following growth of bacterial cells harboring the plasmid, the plasmid DNA was purified from cellular components, concentrated, filtered, and stored until formulation of the drug product. The final vaccine product will meet lot release specifications prior to administration.

The Drug Product is manufactured in phosphate buffered saline (PBS). Vials are aseptically filled to a volume of 1.2 mL with 4 mg/mL of plasmid.

2.2 Formulation, Manufacture, and Packaging of Inactivated H7N9 MIV

Inactivated monovalent split-virion A/Shanghai/2/2013 (H7N9) influenza vaccine was produced by Sanofi Pasteur Inc., Swiftwater, PA. The vaccine reference reassortant A/Shanghai/2/2013 used for vaccine production was provided by the CDC, and the production was completed following the procedures and methods used to manufacture licensed influenza virus vaccines such as Fluzone®. Monovalent bulk concentrate production, bulk formulation of the vaccine, and filling operations were all performed in the licensed facilities. The investigational vaccine is essentially identical to the Fluzone seasonal influenza vaccine, except that it contains only the H7N9 strain. The vaccine was produced in eggs; antibiotics were not used in the manufacturing of the vaccine, and it contains no preservative, no latex or adjuvant. Please refer to the Investigator’s Brochure for details on vaccine production and formulation.

The A/Shanghai/2/2013 (H7N9) MIV vaccine was formulated with phosphate buffered saline at a concentration of HA at 7.5, 15 or 30 mcg per 0.5 mL dose. Only 30 mcg per 0.5 mL individual dose vials will be used in this protocol.

2.3 Summary of preclinical immunogenicity study of VRC-FLUDNA071-00-VP in Mice

Non-clinical, non-GLP immunogenicity studies in mice were conducted at the NIH Vaccine Research Center with VRC-FLUDNA071-00-VP administered intramuscularly and intradermaly with electroporation at 15 mcg dose. The results confirmed that the VRC-FLUDNA071-00-VP Drug Product is immunogenic. As detected by hemagglutinin inhibition (HAI) and neutralization assay, vaccine induced antibody responses conferred protection from lethal challenge with A/Anhui/01/2013(H7N9) influenza.

2.4 Preclinical Studies with H7N9 MIV

No preclinical pharmacology, toxicology, pharmacokinetic, or metabolism studies were conducted for the inactivated A/Shanghai/2/2013 (H7N9) vaccine.

3. STUDY OBJECTIVES

The purpose of this study is to evaluate the safety, tolerability, and immunogenicity of the H7 DNA prime- H7N9 MIV boost vaccination regimen in comparison to the MIV-MIV prime-boost, with a 16 week boost interval for both regimens. For the study objectives, an HAI response (seroconversion) is defined as ≥1:40 titer for baseline-negative subjects with the HAI

titer < 1:10 at Day 0, or 4-fold greater than baseline for subjects with pre-existing immunity (a baseline HAI titer ≥ 1:10).

3.1 PRIMARY OBJECTIVE

- To evaluate the safety and tolerability of three prime-boost vaccine regimens:
  1) H7 DNA vaccine, VRC-FLUDNA071-00-VP, administered at 4 mg IM with the Biojector at Day 0 followed by H7N9 MIV administered at 45 mcg IM with needle injection at Study Week 16;
  2) H7 DNA vaccine administered at 4 mg IM with the Biojector in one arm and MIV administered at 45 mcg IM with needle injection in a different arm as a prime at Day 0 followed by MIV boost at Study Week 16 and
  3) H7N9 MIV administered at 45 mcg IM with needle injection at Day 0 and Study Week 16.

3.2 SECONDARY OBJECTIVES

- To compare the magnitude and the frequency of H7-specific antibody response as measured by HAI at 2 weeks after the MIV boost for each regimen.

3.3 EXPLORATORY OBJECTIVES

- To evaluate the magnitude and the frequency of H7-specific neutralizing antibodies that are 4-fold greater than baseline at 2 weeks after the MIV boost for each group.
- To evaluate the frequency and magnitude of H7-specific T cell responses and antibody responses at specified timepoints throughout the study.

4. STUDY DESIGN AND METHODS

This is a Phase I, randomized, open-label study to evaluate the safety, tolerability, and immunogenicity of prime-boost vaccination regimens against H7N9 Influenza as shown in **Table 2**. A vaccination regimen with the VRC-FLUDNA071-00-VP (H7 DNA) vaccine administered alone or concurrently in different arms with inactivated A/Shanghai/2/2013(H7N9) (MIV) as a prime followed by MIV boost will be compared to MIV-MIV prime-boost with a 16 week boost interval for all schedules. The primary hypothesis is that all vaccination regimens will be safe for human administration. A secondary hypothesis is that the DNA-MIV regimen will elicit a greater frequency and/or magnitude of antibody responses against H7 than the MIV-MIV regimen. The primary objective is to evaluate the safety and tolerability in healthy adults of the investigational vaccines' regimens. Secondary objectives are related to the immunogenicity of the vaccination regimens.

The study will be conducted by the VRC Clinic, NIAID, at a single site at the NIH Clinical Center. The visit schedule is shown in Appendix III. The expected duration of time on study per subject is approximately 28 weeks.

**Table 2. VRC 315 Study Schedule**			
**Group**	**Subjects***	**Day 0**	**Week 16 (±4 weeks)**
Group 1	10	H7 DNA	H7N9 MIV
Group 2	10	H7 DNA+H7N9 MIV	H7N9 MIV
Group 3	10	H7N9 MIV	H7N9 MIV
Total	30	H7 DNA injections are 4 mg administered as 1 mL IM by Biojector. H7N9 MIV injections are 45 mcg administered as 0.75 mL IM by needle/syringe. Prime injections in Group 2 will be administered in different arms.	
*Up to 6 subjects over target enrollment are permitted in the study. Randomizations will be stratified by age; up to 9 subjects ages 51-60 years will be enrolled.			

4.1 STUDY POPULATION

Healthy subjects, ages 18-60 years, will be recruited through Institutional Review Board (IRB)-approved advertising and screened through the VRC 500 (11-I-0164) screening protocol to confirm eligibility requirements for participation.

The following eligibility criteria will be used:

4.1.1 Inclusion Criteria

A subject must meet all of the following criteria:

1. 18 to 60 years old.
2. Available for clinic visits for up to 28 weeks after enrollment.
3. Able to provide proof of identity to the satisfaction of the study clinician completing the enrollment process.
4. Willing to donate blood for sample storage to be used for future research.
5. In good general health without clinically significant medical history.
6. Received a current seasonal influenza vaccine at least 2 weeks before enrollment.
7. Physical examination and laboratory results without clinically significant findings and a Body Mass Index (BMI) ≤40 within the 56 days prior to enrollment.

Laboratory Criteria within 56 days prior to enrollment:

8. Hemoglobin within institutional normal limits
9. White blood cells (WBC) and differential either within institutional normal range or accompanied by site physician approval
10. Total lymphocyte count ≥ 800 cells/mm^3
11. Platelets = 125,000 – 500,000/mm^3
12. Alanine aminotransferase (ALT) ≤ 1.25 x upper limit of normal (ULN)

13. Serum creatinine ≤ 1.1 x ULN based on the institutional normal range

Criteria applicable to women of childbearing potential:

14. Negative human chorionic gonadotropin (β-HCG) pregnancy test (urine or serum) on day of enrollment

15. Agree to use an effective means of birth control from 21 days prior to enrollment through 4 weeks after the second study vaccination

4.1.2 Exclusion Criteria

A subject will be excluded if one or more of the following conditions apply.

Women Specific:

1. Breast-feeding or planning to become pregnant while participating in the study

Subject has received any of the following substances:

2. More than 10 days of systemic immunosuppressive medications or cytotoxic medications within the 4 weeks prior to enrollment or any within the 14 days prior to enrollment

3. Blood products within 16 weeks prior to enrollment

4. Live attenuated vaccines within 4 weeks prior to initial study vaccine administration

5. Investigational research agents within 4 weeks prior to enrollment or planning to receive investigational products while on the study

6. Medically indicated subunit or killed vaccines, e.g. influenza, pneumococcal within 2 weeks of initial study vaccine administration unless approved by the study Principal Investigator (PI)

7. Allergy treatment with antigen injections, unless on maintenance schedule

8. Current anti-TB prophylaxis or therapy

9. Previous H7 avian influenza investigational vaccine

Subject has a history of any of the following clinically significant conditions:

10. Contraindication to receiving an FDA-approved current seasonal influenza vaccination including hypersensitivity to eggs

11. Serious reactions to vaccines that preclude receipt of study vaccinations as determined by the investigator

12. Hereditary angioedema, acquired angioedema, or idiopathic forms of angioedema

13. Asthma that is unstable or required emergent care, urgent care, hospitalization or intubation during the past two years or that requires the use of oral or intravenous corticosteroids

14. Diabetes mellitus (type I or II), with the exception of gestational diabetes

15. Thyroid disease that is not well controlled

16. Idiopathic urticaria within the past year

17. Hypertension that is not well controlled

18. Bleeding disorder diagnosed by a doctor (e.g. factor deficiency, coagulopathy, or platelet disorder requiring special precautions) or significant bruising or bleeding difficulties with IM injections or blood draws

19. Malignancy that is active or treated malignancy for which there is not *reasonable* assurance of sustained cure or malignancy that is likely to recur during the period of the study

20. Seizure disorder other than: 1) febrile seizures, 2) seizures secondary to alcohol withdrawal more than 3 years ago, or 3) seizures that have not required treatment within the last 3 years

21. Asplenia, functional asplenia or any condition resulting in the absence or removal of the spleen.

22. Type 1 hypersensitivity reaction to aminoglycoside antibiotics

23. Guillain-Barré Syndrome

24. Psychiatric condition that precludes compliance with the protocol; past or present psychoses; past or present bipolar disorder; disorder requiring lithium; or within 5 years prior to enrollment, a history of suicide plan or attempt

25. Any medical, psychiatric, social condition, occupational reason or other responsibility that, in the judgment of the investigator, is a contraindication to protocol participation or impairs a volunteer’s ability to give informed consent

4.2 SUBJECT MONITORING: SCHEDULES OF EVALUATIONS

Evaluation of the safety of these vaccines will include laboratory studies, medical history, physical assessment by clinicians, and subject self-assessment recorded on a diary card following each injection. Potential adverse reactions will be further evaluated prior to continuing the immunization schedule. The study schedule is described in **Section 4.2.2** and presented in the form of a Table in **Appendix III**. Total blood volume drawn from each subject will not exceed the NIH Clinical Center Guidelines.

4.2.1 Screening

Screening for this study will be completed through the VRC screening protocol, VRC 500 (NIH 11-I-0164). Testing will be done according to eligibility criteria and clinical assessment at screening. Screening evaluations for specific eligibility criteria (see **Sections 4.1.1** and **4.1.2**) must be completed within the time interval specified prior to enrollment for the given parameter, but may be repeated as needed to confirm eligibility. Storage samples will also be collected during screening; although generally collected in the 56 days prior to enrollment, a particular interval of time prior to enrollment for collection of these samples is not specified. Informed consent documents for vaccine trials will be reviewed, and counseling relating to the potential risks of becoming pregnant during study participation will be provided. Any study volunteer who has not yet received the current year influenza vaccination may receive this, if available, during participation in the screening protocol. An Assessment of Understanding (AoU) will be completed in association with enrollment into VRC 315.

4.2.2 Study Schedule and Randomization

Day 0 is defined as the day of protocol enrollment and first injection. Protocol-specific eligibility is reviewed on Day 0 as part of the enrollment process, but eligibility evaluations conducted during a screening visit (VRC 500) are routinely used for eligibility if the screening occurred within the specified window prior to the Day 0 visit. Pregnancy test results for women of reproductive potential must be obtained on each injection day prior to the study injection. Day 0 evaluations prior to the first injection are the baseline for subsequent safety assessments.

All vaccinations will be administered according to the randomization assignment to one of the study groups. Neither the clinic staff nor the subjects will know in advance which group will be assigned to the enrollees in the study. The Protocol Statistician will prepare the randomization plan in advance and provide it to the Site Pharmacy and Data Management Center. The pharmacy database and study randomization will be set up prior to opening the study to accrual. The group assignment will become known to the staff and subject immediately after completing the electronic enrollment into the study on Day 0. The visit schedule is the same for all groups, and it is based on intervals of time after each study injection (see **Appendix III**). Any subject who receives at least one study injection will continue with follow-up for at least 12 weeks of safety follow-up. Otherwise, follow-up will continue through 12 weeks after the Study Week 16 booster injection; thus, the last required clinic visit will be Study Week 28.

The schedule of study visits, permitted windows for completing the visits, and evaluations performed at each visit are shown in **Appendix III**. After Day 0, deviations from the visit windows in completing study visits and study injections are discouraged and will be recorded as protocol deviations, but are permitted at the discretion of the PI (or designee) in the interest of completing the vaccination schedule and obtaining subject safety and immunogenicity evaluations.

4.2.3 Administration of Injections

On each injection day (prior to injection) study subjects will be evaluated by clinical evaluation and samples will be collected for laboratory tests as per Schedule of Evaluations. A subject who arrives at the clinic with fever or evidence of an acute illness, which precludes administration of the vaccine, may be rescheduled within the allowed visit window.

It is recommended, but not required, that the first injection be administered into the non-dominant arm. It is preferred, but not required, to alternate arms for study injections. When choosing an arm for the injection, clinicians should consider whether there is an arm injury, local skin problem or significant tattoo that precludes administering the injection or will interfere with evaluating the arm after injection.

Group 2 participants will receive the H7 DNA and H7N9 MIV as two injections administered in different arms on Day 0.

All MIV injections will be administered IM at a dosage of 45 mcg into the deltoid muscle by needle and syringe.

All H7 DNA vaccine injections will be administered IM into the deltoid muscle at a dosage of 4 mg using a Biojector 2000® needle-free injection system (Biojector; Bioject Medical Technologies Inc., Tigard, OR).

Following each study injection, subjects will be observed for a minimum of 30 minutes. Vital signs (temperature, blood pressure, pulse and respiratory rate) will be taken at 30 minutes or longer after the injection, prior to departure from the clinic. The injection site(s) will be inspected for evidence of local reaction. In keeping with the NIH Clinical Center policy and good medical practice, acute medical care will be provided to subjects for any immediate allergic reactions or other injury resulting from participation in this research study.

4.2.4 Diary Cards and Follow-up

Subjects will be given a "Diary Card" to use as a memory aid, on which to record temperature and symptoms daily for 7 days after each injection. Subjects will be trained to use the secure database or complete the paper diary card depending on their preference. When the diary card parameters are recorded directly by the subject through a password-protected secure database, the subject's electronic record will be the source for these data. The written (paper) diary card may be used as a source document. When neither a written nor electronic diary card is available from the subject, the study clinician will note the source of reactogenicity information recorded in the study database.

The solicited signs and symptoms on the diary card will include the parameters: unusually tired/feeling unwell, muscles aches (other than at injection site), headache, chills, nausea, joint pain, and pain/tenderness at injection site. Subjects will also record the day's highest measured temperature and measurement of largest diameter for redness and swelling at injection site.

Follow-up on subject well-being will be performed as shown in the Schedule of Evaluations (**Appendix III**). Events following any study injection that may require clinical evaluation include rash, urticaria, fever of 38.5˚C (Grade 2) or higher lasting greater than 24 hours, or significant impairment in the activities of daily living. Additionally, other clinical concerns may prompt a study visit based on the judgment of a study clinician.

4.2.5 Blood Sample Collection

At intervals throughout the study, blood will be drawn for safety and immunologic assays. Blood will be drawn from the arm veins of subjects by standard phlebotomy procedures.

Apheresis: Apheresis will be offered as an optional procedure at Visit 06A. Visit 06A may occur on the same day or a different day as Visit 06 since these visits have overlapping, but different

preferred visit windows. Each apheresis procedure will be carried out by trained DTM medical staff using automated cell separator devices. In this study, the procedure will require one or two antecubital venous access sites and will involve processing one to four liters of whole blood. The expected mononuclear cell yield is approximately 0.5 to 1.0 x 10^9 cells per liter processed, and the apheresis device can process about 2-3 liters per hour. Thus, 1 to 2 hours are required to process 1 to 4 liters of blood and obtain about 1 - 4 x 10^9 leukocytes. The packed red cell loss during the procedure is the equivalent of a 6 mL blood draw; this is the volume that will be used for the purposes of calculating cumulative blood draw when apheresis is performed. A fingerstick hemoglobin assessment will be performed prior to initiating apheresis, per DTM Apheresis Clinic standard policies.

Blood for research will be processed and samples stored at NVITAL. Stored samples may be used later to further evaluate immune responses and to elucidate genetic factors associated with immune response.

4.2.6 Concomitant Medications

Current concomitant medications will be recorded in the study database at enrollment. Concomitant medications will be updated in the study database if there is an occurrence of an adverse event that requires expedited reporting. Whether or not any medications were taken during the 7-day period following vaccination will be documented. Treatment for influenza with antiviral drugs will be recorded on an Influenza Endpoint Case Report Form.

Clinicians should work with study subjects with regard to the timing of FDA-approved vaccines. Intercurrent vaccinations with licensed vaccines do not require discontinuation of the vaccination schedule; however, the Principal Investigator has the option of discontinuing the vaccination schedule or allowing some flexibility in the subsequent study injection based on clinical judgment and the potential effect on the immunogenicity analysis. Receipt of a licensed vaccine at any time is not a protocol violation. Vaccinations received during study participation will be recorded in the study database. Otherwise, a record of concomitant medication changes throughout the study will not be recorded in the study database.

4.3 CRITERIA FOR DISCONTINUING STUDY INJECTIONS OR PROTOCOL PARTICIPATION

In general, subjects who receive the Day 0 study injection will continue to be followed for at least 12 weeks of safety follow-up, whenever possible. Decisions on discontinuation of study injections or protocol participation for a subject will be made by the study PI or designee.

4.3.1 Discontinuation of Study Injections

Under certain circumstances, a subject will be terminated from participating in further injections. Participants who are discontinued from additional study injections will continue to be followed according to the schedule of safety and immunogenicity evaluations. Specific events that will require withdrawal of a subject from the injection schedule include:

1. Pregnancy;
2. Grade 2 adverse event assessed as related to a study injection that does not resolve to baseline in time for the next scheduled immunization;
3. Grade 3 adverse event assessed as related to a study injection (with the exception that

self-limited Grade 3 solicited reactogenicity does not require discontinuation of study injections);

4. Grade 4 adverse event assessed as related to a study injection;
5. Immediate hypersensitivity reaction associated with a study injection;
6. Intercurrent illness that is not expected to resolve prior to the next scheduled study injection assessed by study clinician to require withdrawal from the injection schedule;
7. Treatment with systemic glucocorticoids (e.g., prednisone or other glucocorticoid) or other immunomodulators (other than nonsteroidal anti-inflammatory drugs [NSAIDs]), with the exception that, study vaccination may continue per investigator discretion if the next one occurs at least 2 weeks following completion of glucocorticoid treatment.
8. The study PI assesses that it is not in the best interest of the subject to continue on the vaccination schedule.

4.3.2 Discontinuation from Protocol Participation

A subject may be discontinued from protocol participation for the following reasons:

1. Subject decides to discontinue participation.
2. Subject develops a medical condition that is a contraindication to continuing study participation.
3. The IND sponsor or regulatory authority stops the protocol.
4. The study PI assesses that it is not in the best interest of the subject to continue participation in the study or that the subject's compliance with the study is not sufficient.

4.4 CRITERIA FOR PAUSING THE STUDY

The Principal Investigator will closely monitor and analyze study data as they become available and will make determinations regarding the presence and severity of adverse events. The administration of study injections and new enrollments will be paused and the IND Sponsor will be promptly notified according to the following criteria:

- **One** (or more) subject experience a Serious Adverse Event (SAE) assessed as related to study vaccination, or an SAE that does not have a clear, attributable cause.
- **Two** (or more) subjects experience the same **Grade 3** or higher unsolicited adverse event assessed as related to the same study vaccine, or that does not have a clear, attributable cause. (Notes: adverse events occurring after the H7 DNA vaccine will be counted separately from those occurring after the H7N9 MIV vaccine; also, self-limited solicited reactogenicity will not be counted towards pause criteria).

Plan for Review of Pauses and Resuming Rules:

The study injections and enrollments would resume only if review of the adverse events that caused the pause resulted in a recommendation to permit further study injections and study enrollments. The reviews to make this decision will occur as follows:

Pauses for SAEs: The IND Sponsor, with participation by the Principal Investigator, will consult with the FDA to conduct the review and make the decision to resume or close the study for any SAEs that meet the criteria for pausing the study.

Pauses for Grade 3 or Higher Adverse Events: The IND Sponsor, in consultation with the Principal Investigator, will conduct the review and make the decision to resume or close the study for the adverse events that meet the criteria for pausing the study. As part of the pause review, the reviewers will also advise on whether the study needs to be paused again for any subsequent adverse events of the same type.

When indicated, safety data reports and changes in study status will be submitted to the IRB in accordance with **Section 5.4** and institutional policy.

5. SAFETY AND ADVERSE EVENT REPORTING

5.1 ADVERSE EVENTS

An adverse event (AE) is any untoward or unfavorable medical occurrence in a human subject, including any abnormal sign (e.g., abnormal physical exam or laboratory finding), symptom, or disease temporally associated with the use of study treatment, whether or not considered related to the study treatment.

Each adverse event will be graded according to the table for grading severity of adverse events (see **Appendix IV**). The following guidelines will be used to determine whether or not an adverse event is recorded in the study database:

Solicited adverse event (i.e, reactogenicity parameters) will be recorded in the study database for 7 days after each injection without the collection of attribution assessments. All unsolicited AEs will be recorded in the study database from receipt of first study injection through the visit scheduled for "4 weeks" after each study injection. At other time periods between injections and when greater than 4 weeks after the booster MIV injection, only SAEs (as detailed in **Section 5.2**), new chronic medical conditions, and influenza or influenza-like illness will be recorded through the last study visit. Cases of influenza or influenza-like illness will be evaluated using clinical judgment and current CDC definition of influenza symptoms (http://www.cdc.gov/flu/about/disease/symptoms.htm), and recorded on an influenza endpoints form rather than on an adverse events form.

5.2 SERIOUS ADVERSE EVENTS

As defined in 21 CFR 312.32, an adverse event or suspected adverse reaction is considered "serious" if, in the view of either the investigator or sponsor, it results in any of the following outcomes: "Death, a life-threatening adverse drug experience, inpatient hospitalization or prolongation of existing hospitalization, a persistent or significant disability/incapacity, or a congenital anomaly/birth defect. Important medical events that may not result in death, be life-threatening, or require hospitalization may be considered a serious adverse drug experience when, based upon appropriate medical judgment, they may jeopardize the subject or require medical or surgical intervention to prevent one of the outcomes listed in this definition. Examples of such medical events include allergic bronchospasm requiring intensive treatment in an emergency room or at home, blood dyscrasias or convulsions that do not result in inpatient hospitalization, or the development of drug dependency or drug abuse."

"Life threatening" refers to an adverse event that at occurrence represented an immediate risk of death to the subject; it does not refer to an event that hypothetically might have caused death if it were more severe. Similarly, a hospital admission for an elective procedure is not considered a Serious Adverse Event.

5.3 Adverse Event Reporting to the IND Sponsor

Adverse events that meet Serious Adverse Event (SAE) Reporting Requirements must be reported and submitted by the clinical site on an expedited basis to the IND sponsor, VRC/NIAID/NIH, according to sponsor guidelines as follows:

- results in death
- is life threatening
- results in persistent or significant disability/incapacity
- requires unplanned inpatient hospitalization or prolongation of existing hospitalization
- is a congenital anomaly/birth defect in the offspring of a study subject
- is an important medical event that may jeopardize the subject or may require intervention to prevent one of the other outcomes listed above.

In addition, any event, regardless of severity, which in the judgment of an investigator represents a serious adverse event, may be reported on an expedited basis.

An investigator will communicate an initial SAE report within 24 hours of site awareness of occurrence to the IND sponsor by email to the VRC Protocol Operations Office (see **Appendix II**).

A written report by investigator should be submitted to the IND Sponsor within 3 working days. In order for the IND Sponsor to comply with regulations mandating sponsor notification of specified SAEs to the FDA within 7 or 15 calendar days, the investigator must submit additional information as soon as it is available.

5.3.1 IND Sponsor Reporting to the FDA

It is the responsibility of the IND Sponsor to make the determination of which SAEs are “serious and unexpected suspected adverse reactions” (SUSARs) as defined in 21 CFR 312.32.

- *Suspected adverse reaction* means any adverse event for which there is a reasonable possibility that the drug caused the adverse event.
- *Unexpected Adverse Event* means an AE that is not listed in the Investigator’s Brochure or is not listed at the specificity or severity that has been observed.

All SUSARs, as determined by the IND Sponsor, will be reported to the FDA as IND Safety Reports and IND Safety Reports will be provided to the IRB.

The IND Sponsor will also submit an IND Annual Report of the progress of the investigation to the FDA as defined in 21 CFR 312.33.

Copies of all FDA reports will be provided to the H7N9 MIV manufacturer, Sanofi Pasteur, Inc.

5.4 Reporting to the Institutional Review Board

5.4.1 Unanticipated Problem (UP) Definition

A serious “Unanticipated Problem (UP)” is defined as any incident, experience, or outcome that meets all three of the following criteria:

- unexpected in nature, severity, or frequency in relation to the research risks that are described in the protocol, informed consent, Investigator’s Brochure, other study

documents or in consideration of the characteristics of the subject population being studied; **and**

- related to participation in the research; **and**
- suggests that the research places subjects or others at a greater risk of harm (including physical, psychological, economic, or social harm) than was previously known or recognized.

Non-serious UP: An UP that is not an Adverse Event (UPnonAE) is an unanticipated problem that does not fit the definition of an adverse event, but which may, in the opinion of the investigator, involve risk to the subject, affect others in the research study, or significantly impact the integrity of research data. Such events would be considered a non-serious UP. For example, we will report occurrences of breaches of confidentiality, accidental destruction of study records, or unaccounted-for study drug.

5.4.2 Protocol Deviation Definition

A Protocol Deviation is defined as any change, divergence, or departure from the IRB-approved study procedures in a research protocol. Protocol deviations are designated as serious or non-serious and further characterized as

- Those that occur because a member of the research team deviates from the protocol.
- Those that are identified before they occur, but cannot be prevented.
- Those that are discovered after they occur.

Serious Protocol Deviation: A deviation that resulted in a Serious Adverse Event or compromises the safety, welfare or rights of subjects or others.

5.4.3 Non-Compliance Definition

Non-compliance is the failure to comply with applicable NIH Human Research Protections Program (HRPP) policies, IRB requirements, or regulatory requirements for the protection of human subjects. Non-compliance is further characterized as serious, continuing or minor.

“Serious non-compliance” is defined as non-compliance that

- Increases risks, or causes harm, to participants
- Decreases potential benefits to participants
- Compromises the integrity of the NIH-HRPP
- Invalidates the study data

“Continuing non-compliance” is non-compliance that is recurring.

“Minor non-compliance” is non-compliance that is neither serious nor continuing.

5.4.4 Expedited Reporting to the NIAID IRB

The following will be reported within 7 calendar days of investigator awareness:

- Serious and non-serious UP
- Deaths
- Serious protocol deviations

- Serious or continuing non-compliance
- SAEs that are possibly, probably, or definitely related to the research regardless of expectedness

The following waiver applies to reporting anticipated protocol deviations and expected UPnonAEs: Anticipated deviations in the conduct of the protocol will not be reported to the IRB unless they occur at a rate greater than anticipated by the study team. Expected adverse events will not be reported to the IRB unless they occur at a rate greater than that known to occur in healthy adults. If the rate of these events exceeds the rate expected by the study team, the events will be classified and reported as though they are unanticipated problems.

5.4.5 Annual Reporting to the NIAID IRB

The following will be reported to the NIAID IRB in summary at the time of Continuing Review:

- Serious and non-serious UP
- Expected SAEs that are possibly, probably, or definitely related to the research
- SAEs that are not related to the research
- All adverse events, except expected AEs granted a waiver of reporting
- Serious and Non-Serious Protocol Deviations
- Serious, continuing, and minor non-compliance
- Any trends or events which in the opinion of the investigator should be reported

5.5 Serious Adverse Event Reporting to the Institutional Biosafety Committee

The NIH Institutional Biosafety Committee (IBC) (Building 13, Room 3K04, NIH, Bethesda, MD) has the responsibility to review research using recombinant DNA for compliance with NIH Guidelines. In keeping with IBC requirements, any SAE reports sent to the IRB will be subsequently provided to the IBC by the investigators.

6. STATISTICAL CONSIDERATIONS

6.1 Overview

This study is a single-center trial to assess the safety and tolerability of 3 prime-boost schedules as per study schema (**Table 2**). An assessment of immunogenicity will also be performed.

6.2 Objectives

The primary objective of this trial concerns safety. The secondary and exploratory objectives concern immunogenicity.

6.3 Endpoints

6.3.1 Primary Endpoints: Safety

Assessment of product safety will include clinical observation and monitoring of hematological and chemical parameters. Safety will be closely monitored after injection and evaluated through 12 weeks after last study injection. See **Section 4.2** and **Appendix III** for details and specified time points. The following parameters will be assessed for all study groups:

- Occurrence of solicited local reactogenicity signs and symptoms for 7 days following each vaccination

- Occurrence of solicited systemic reactogenicity signs and symptoms for 7 days following each vaccination
- Change from baseline for safety laboratory measures
- Occurrence of adverse events of all severities through 4 weeks after the prime injection and through 4 weeks after MIV boost
- Occurrence of serious adverse events through last study visit
- Occurrence of influenza or influenza-like illness events through last study visit

6.3.2 Secondary Endpoints: Immunogenicity

The primary immunogenicity endpoint is H7-specific antibody responses as measured by H7 HAI. The main timepoints are Week 0 (baseline) and 2 weeks after the boost.

For all immunogenicity endpoints, seroconversion is defined as the proportion of subjects with either a pre-vaccination H7-specific HAI titer <1:10 and a post-vaccination HAI titer ≥1:40 or a pre-vaccination HAI titer ≥1:10 and a minimum four-fold rise in post-vaccination HAI antibody titer.

6.3.3 Exploratory Endpoints

The magnitude and the frequency of H7-specific neutralizing antibodies that are 4-fold greater than baseline at 2 weeks after the MIV boost for each group will be evaluated.

Evaluation of antibody and T cell responses at time points throughout the study will be completed as an exploratory evaluation.

For the H7 neutralization assay, a positive response for a subject is defined as the subject achieving a four-fold rise in the H7-specific neutralization antibody titer from baseline.

6.4 SAMPLE SIZE AND ACCRUAL

The study design is to have 30 healthy adult participants divided equally among 3 schedules.

6.4.1 Power Calculations for Safety

The goal of the safety evaluation for this study is to identify safety concerns associated with injections of the investigational vaccines. Primary sample size calculations for safety are expressed in terms of the ability to detect serious adverse experiences. Other sample size calculations for comparing of the two vaccination groups on adverse experiences are similar to the calculations for immunogenicity (see **Section 6.4.3**).

The ability of the study to identify SAEs will be expressed in terms of the probability of observing a certain number of serious adverse events. Useful values are the minimum true rate such that the probability of observing at least one event is at least 90%, and the maximum true rate such that the probability of not observing any event is at least 90%. Within each group (n=10), there is over 90% chance to observe at least 1 SAE if the true rate is at least 0.206 and over 90% chance to observe no SAE if the true rate is no more than 0.01.

Probabilities of observing 0 or more than 1 serious adverse event within each group are presented in **Table 6-1** for a range of possible true event rates. These calculations provide a more complete picture of the sensitivity of this study design to identify potential safety problems with

the vaccine.

Table 6-2 gives the upper and lower bounds for 95% exact binomial confidence intervals of the true SAE rate at all possible numbers of events within each group (n=10). If none of the 10 vaccinees experience serious adverse events, the 95% exact 2-sided upper confidence bound for the SAE rate is 0.308.

Table 6-1: Probability of Events for Different Safety and Immunogenicity Scenarios within a Group (n=10)

True Event rate	Pr (observing 0 event)	Pr (observing more than 1 event)
0.005	0.951	0.001
0.010	0.904	0.004
0.020	0.817	0.016
0.035	0.700	0.046
0.050	0.599	0.086
0.100	0.349	0.264
0.150	0.197	0.456
0.200	0.107	0.624
0.300	0.028	0.851

Table 6-2: 95% Confidence Intervals for the True Rate At All Possible Observed Rates within a Group (n=10)

	95% Confidence Interval	
Observed Rate	Lower Bound	Upper Bound
0/10	0	0.308
1/10	0.003	0.445
2/10	0.025	0.556
3/10	0.067	0.652
4/10	0.122	0.738
5/10	0.187	0.813
6/10	0.262	0.878
7/10	0.348	0.933
8/10	0.444	0.975
9/10	0.555	0.997
10/10	0.692	1

6.4.2 Sample Size Calculations for Immunogenicity

Table 6-2 is applicable to the immunogenic response rates, and gives the exact 95% confidence interval of the true response rate over possible number of responses out of the 10 subjects. For example, if we observe 5 responses among the 10 vaccinees, the 95% exact binomial confidence interval of the true response rate will range from 0.187 to 0.813.

Table 6-1 gives the probabilities of observing 0 or at least 2 responses over a range of underlying response rates. For example, if the true response rate at a particular time point is 0.20, then there is a probability of 0.893 to observe at least one response and a probability of 0.624 to observe at least two responses among the 10 vaccinees in a group.

6.4.3 Power for Comparison

To compare the two schedules for possible difference in immunogenicity, a simple comparison on the positive response rate will be used. **Table 6-3** gives the power of Fisher exact test to compare any two schedules over a range of possible response rates. It indicates that a comparison between two groups is not powered by the study design with 10 samples per group.

Table 6-3: Power to Detect Difference in Response Rates between Two groups by Fisher's Exact Test, Each Group of Size n=10

		Arm 1 Rate (n=10)			
Arm 2 Rate (n=10)		0.2	0.3	0.4	0.5
	0.1	1	5	15	29
	0.2		2	5	13
	0.5	13	5	2	
	0.6	25	12	5	2

With 10 subjects per group, there is 80% power to detect between-group difference in magnitude of effect size (the ratio of difference to standard deviation) 1.33 or higher. For ELISA and neutralization data, if both groups have log(e)-transformed data of standard deviation 1, the sample size allows for detection of between-group difference of 1.33 or higher in the log-transformed Geometric Mean Titers (GMTs). This difference of 1.33 in log-scale translates to about a 4-fold difference (exp(1.33)=3.78) in GMT.

6.5 STATISTICAL ANALYSIS

Since enrollment is concurrent with receiving the first study vaccination, the expectation is that all participants will receive at least one vaccination and therefore will provide some safety data.

All statistical analyses will be performed using Statistical Analysis System (SAS), R, or S-Plus statistical software.

No formal multiple comparison adjustments will be employed for safety endpoints or secondary endpoints.

6.5.1 Analysis Variables

The analysis variables consist of baseline variables, safety variables, and immunogenicity variables for primary and secondary objective analyses.

6.5.2 Baseline Demographics

Baseline characteristics including demographics and laboratory measurements will be summarized using descriptive statistics.

6.5.3 Safety Analysis

Reactogenicity

The number and percentage of participants experiencing each type of reactogenicity sign or symptom will be tabulated by severity. For a given sign or symptom, each participant's reactogenicity will be counted once under the maximum severity for all assessments.

Adverse Experiences

Adverse experiences are coded into Medical Dictionary for Regulatory Activities (MedDRA) preferred terms. The number and percentages of participants experiencing each specific adverse event will be tabulated by severity and relationship to treatment. For the calculations in these tables, each participant's adverse experience will be counted once under the maximum severity or strongest recorded causal relationship to treatment.

A complete listing of adverse experiences for each participant will provide details including severity, relationship to treatment, onset, duration and outcome.

Local Laboratory Values

Boxplots of local laboratory values will be generated for baseline values and for values measured during the course of the study. Each boxplot will show the 1st quartile, the median, and the 3rd quartile. Outliers, or values outside the boxplot, will also be plotted. If appropriate, horizontal lines representing boundaries for abnormal values will be plotted.

6.5.4 Immunogenicity Analysis

The statistical analysis for immunogenicity will employ the intent-to-treat principle, i.e., all data from enrolled participants will be used. In the final analysis of immunogenicity, if there are cases of a subject receiving a schedule different from the assignment, then an as-treated analysis will be performed.

If assay data are qualitative (i.e., positive or negative) then analyses will be performed by tabulating the frequency of positive response for each assay at each time point that an assessment is performed. Binomial response rates will be presented with their corresponding exact 95% confidence interval estimates. Fisher's exact tests will be used to compare the two vaccine groups to each other. Missing responses will be assumed to be missing at random, i.e., conditional on the observed data the missingness is independent of the unobserved responses. Graphical descriptions of the longitudinal immune responses will also be given.

Some immunologic assays have underlying continuous or count-type readout that is often dichotomized into responder/nonresponder categories. For these assays, graphical and tabular summaries of the underlying distributions will be made. These summaries may be performed on transformed data (*e.g.*, log transformation) to better satisfy assumptions of symmetry and homoscedasticity.

6.5.5 Interim analyses

Safety Reviews: The Protocol Safety Review Team (PSRT) will review safety data routinely throughout the study. The study will utilize both electronic database features and reviews by designated safety review personnel to identify in a timely manner if any of the safety pause rules of the study are met.

Immunogenicity Review: The analyses of immunogenicity may be performed when the HAI assays of samples collected at 4 weeks after the boost have been completed. This may occur

prior to completion of safety follow-up visits or collection of data for secondary and exploratory immunogenicity endpoints. Such an analysis would constitute the final analysis for the primary immunogenicity endpoint, so sample size adjustments are not required. Reports providing results by study group may be provided to VRC solely for the purpose of informing decisions related to future trials in a timely manner. The results should in no way influence the conduct of the VRC 315 trial in terms of early termination or later safety or immunogenicity endpoint assessments. Analyses of secondary and exploratory immunogenicity assays may also be performed as data become available.

6.5.6 Randomization of Treatment Assignments

The randomization sequence will be obtained by computer-generated random numbers and provided to the study pharmacist by the protocol statistician. The provision for enrollment of up to 6 additional subjects will be taken into account in the randomization code development. The randomizations will be stratified by age; up to 9 subjects ages 51-60 years will be enrolled in the study.

The study identifier is assigned after completion of the enrollment form in the electronic study database. To decrease the potential for participant dropouts during the period between randomization and initial vaccination, randomization will occur on Day 0 after the study consent is signed and eligibility is confirmed.

The pharmacist and the statistician are responsible for maintaining security of the treatment assignments. Discussion of the treatment assignment between the VRC clinicians and the pharmacy staff is prohibited until after the enrollment is completed and the randomization becomes known to all.

7. PHARMACY AND VACCINE ADMINISTRATION PROCEDURES

The study groups and vaccination schedules are shown in **Table 4.1** in **Section 4**. Refer to **Section 2** for information about manufacturing of study agents.

7.1 STUDY AGENTS

In this study, the study agents are two investigational vaccines as follows:

- Monovalent influenza subvirion vaccine A/Shanghai/02/2013 is provided at concentration 30 mcg/0.5mL (H7N9 MIV)
- VRC-FLUDNA071-00-VP 4 mg/mL: (H7 DNA vaccine)

7.2 STUDY AGENT PRESENTATION AND STORAGE

7.2.1 Study Agent Labels

The labels for both the H7N9 MIV and VRC-FLUDNA071-00-VP (H7 DNA vaccine) will have specific product information (e.g., part number, lot number, fill volume, storage temperature) included on the product vial labels. The label will contain an Investigational Use Statement (“Caution: New Drug – Limited by Federal Law to Investigational Use”), and manufacturer information.

7.2.2 Study Agent Storage:

H7N9 MIV: The vaccine will be stored in a refrigerator for which the normal range is 2°C to 8°C. Do not freeze. If deviations in storage temperature occur from the normal allowance for the pharmacy refrigerator, the site pharmacist must report the storage temperature excursion promptly to the PI and the IND sponsor. The excursion must be evaluated and investigated and action must be taken to restore and maintain the desired temperature limits. Pending the outcome of the investigation, the IND sponsor will notify the pharmacist if continued clinical use of the product is acceptable.

VRC-FLUDNA071-00-VP: Upon release by VRC, the DNA vaccine vials will be shipped within the recommended temperature range using appropriate shipping configurations, to the study pharmacist. Vials of the vaccine should be stored until use at -35°C to -15ºC in a qualified, continuously monitored, temperature-controlled freezer. Temperature excursions will be reported per pharmacy guidelines. Storage below -45ºC is not permitted because of the stopper limitation.

If deviations in storage temperature occur from the normal allowance for the pharmacy freezer, the site pharmacist must report the storage temperature excursion promptly to the PI and IND sponsor. The excursion must be evaluated and investigated and action must be taken to restore and maintain the desired temperature limits. Pending the outcome of the investigation, the IND sponsor will notify the pharmacist if continued clinical use of the product is acceptable.

7.3 PREPARATION OF STUDY AGENT FOR INJECTION

This section describes how the site pharmacist will prepare study injections. Clinician instructions on how to select an arm and administer the injection are in **Section 4.2.2**.

H7N9 MIV: The inactivated H7N9 vaccine is supplied in 3 mL vials filled to contain no less than 0.5 mL extractable volume. Vials are intended for single use only. Refer to the group assignment for the study subject. For visits in which the inactivated H7N9 vaccine is to be administered, remove 2 vials of the H7N9 MIV from the refrigerator.

Inspect vials visually for particulate matter and/or discoloration. If either of those conditions exists, the vaccine should not be administered. Gently invert both vials 5 to 7 times to mix. After mixing, the contents should be essentially clear and colorless to opalescent.

An individual syringe with a 0.75 mL volume will be prepared from the 2 vials (30 mcg/0.5 mL each) by the Clinical Center pharmacy such that a total vaccine amount is 45 mcg, and labeled with the subject identifier for transport to the clinic. The pharmacy will also label with information about date and time after which the preparation may not be used. When preparing a dose in a syringe for administration, consideration should be given to the volume of solution that may remain in the needle after the dose is administered. All injections must be administered within 2 hours after removing the vaccine vial from the refrigerator.

VRC-FLUDNA071-00-VP: The DNA vaccine is supplied as a 2 mL glass vial containing a clear colorless isotonic sterile solution. Each vial contains 20% over the amount to be injected in the cGMP grade phosphate-buffered saline. Vials are intended for single use only and do not contain a preservative. They should not be refrozen after thawing. Each vial (4 mg/mL) contains a volume of 1.2 mL (4.8 mg).

Refer to the group assignment for the study subject. For visits in which the DNA vaccine is to be administered, remove a vial of the DNA vaccine 4 mg/mL from the freezer. Allow the vial to

equilibrate to room temperature (15 to 30º C). Swirl the contents gently. Using aseptic technique, withdraw 1 mL of the DNA vaccine from the vial into the Biojector syringe, remove all air bubbles, and cap the syringe. Label the syringe with the subject identifier and the date and time allowance for administration.

One 1 mL injection of the 4 mg/mL preparation will be administered for each 4 mg dose. A dose of vaccine will be prepared in the Clinical Center pharmacy and the prepared Biojector syringe labeled with the subject identifier will be delivered to the clinic for administration. The pharmacy will also label with information about date and time after which the preparation may not be used. All injections must be administered within 8 hours after removing the vaccine vial from the freezer.

7.4 STUDY AGENT ACCOUNTABILITY

7.4.1 Documentation

The study pharmacist will be responsible for maintaining an accurate record of the codes, inventory, and an accountability record of vaccine supplies for this study. Electronic documentation as well as paper copies will be used.

7.4.2 Disposition

The empty vials and the unused portion of a vial will be discarded in a biohazard containment bag and incinerated or autoclaved following the injection. Any unopened vials that remain at the end of the study will be discarded at the discretion of the VRC in accordance with policies that apply to investigational agents. Partially used vials or expired prepared doses cannot be administered to other subjects nor used for *in vitro* experimental studies and will be discarded as indicated above.

8. HUMAN SUBJECT PROTECTIONS AND ETHICAL OBLIGATIONS

This research study will be conducted in compliance with the protocol, Good Clinical Practices (GCP) guidelines, and all applicable regulatory requirements.

8.1 INSTITUTIONAL REVIEW BOARD

A copy of the protocol, proposed informed consent form, other written subject information, and any proposed advertising material will be submitted to the NIAID IRB for written approval prior to implementation.

The investigator must submit and, where necessary, obtain approval from the IRB for all subsequent protocol amendments and changes to the informed consent document. The investigator will notify the IRB of deviations from the protocol and serious adverse events as per IRB policy.

The investigator will be responsible for obtaining IRB approval of the annual Continuing Review throughout the duration of the study.

8.2 SUBJECT RECRUITMENT AND ENROLLMENT

Subjects for this study will be recruited in accordance with the IRB standard for recruitment practices. Effort will be made to include women and minorities in proportions similar to that of the community from which they are recruited.

8.3 INFORMED CONSENT

The study informed consent is provided in Appendix I. The study consent describes the investigational products to be used and all aspects involved in protocol participation.

Before a subject may participate in the study, it is the investigator's responsibility to obtain written informed consent from the subject, after adequate explanation of the aims, methods, anticipated benefits, and potential hazards of the study and before any protocol-specific procedures or study medications are administered.

The acquisition of informed consent will be documented in the subject's medical records, as required by 21 CFR 312.62, and the informed consent form will be signed and personally dated by the subject and by the person who conducted the informed consent discussion. The original signed informed consent form will be retained in the medical chart and a copy of the informed consent form will be provided to the subject.

8.4 SUBJECT CONFIDENTIALITY

The investigator must ensure that the subject's anonymity is maintained. Subjects will not be identified in any reports on this study. All records will be kept confidential to the extent provided by federal, state and local law. Medical records are made available for review when required by the FDA or other authorized users, such as the vaccine manufacturer, only under the guidelines set by the Federal Privacy Act. Direct access includes examining, analyzing, verifying, and reproducing any records and reports that are important to the evaluation of the study. The investigator is obligated to inform the subjects that the above named representatives will review their study-related records without violating the confidentiality of the subjects. Stored study research samples are labeled by a code (such as a number) that only the VRC Clinic team can link to the subject. The requirement to maintain subject confidentiality is included in the study informed consent document.

8.5 RISKS AND BENEFITS

8.5.1 Risks of the Monovalent Inactivated H7N9 Vaccine

The manufacturing process for the production of the investigational monovalent inactivated A/Shanghai/2/2013 (H7N9) vaccine is similar to the process used to produce the licensed influenza vaccine Fluzone® family of products. Therefore, the safety profile of H7N9 MIV is expected to be similar to the current Fluzone® vaccine.

Occasionally, adult recipients of the licensed inactivated influenza vaccines may develop influenza-like reactions such as fever, body aches, headache, malaise, myalgia and/or nausea. These reactions are usually greatest within the first 24 hours after vaccination and last 1 to 2 days. Some subjects may develop reactions at the site of vaccination (redness, swelling, pain, or tenderness). Analgesics (e.g., aspirin or acetaminophen) and rest will generally relieve or moderate these symptoms. These reactions should go away in 1 to 4 days and not require additional treatment.

Acute and potentially life-threatening allergic reactions are also possible. Since the H7N9 MIV contain limited quantity of egg protein, this protein can induce immediate hypersensitivity reactions among person who have severe allergy. Allergic reactions include hives, angioedema, allergic asthma, and systemic anaphylaxis.

During the swine influenza vaccine campaign of 1976, about 1 per 100,000 vaccine recipients developed a paralytic illness called Guillain-Barré Syndrome. This has not been seen consistently with other influenza vaccines. Most persons who develop Guillain-Barré recover completely.

As of May15, 2014 in other clinical trials with the same H7N9 MIV, the IND Sponsor (DMID/NIAID) has assessed one event as a serious unexpected suspected adverse reaction (SUSAR). This was an acute myocardial infarction (MI) in a 52 year old male subject 4 days after vaccination. Other SAEs to date have been assessed by the IND sponsor as not related to study product.

There may be other unknown side effects.

8.5.2 Risks of the H7 DNA vaccine

This is the first study in humans of the investigational DNA vaccine, VRC-FLUDNA071-00-VP. The risks noted are based on risks from the earlier DNA vaccine studies of similar influenza DNA vaccines, as well as risks of vaccines in general, and results of previous studies with other investigational DNA vaccines.

Potential side effects resulting from intramuscular injection include stinging, arm discomfort, redness of the skin, mild bruising or a small laceration at vaccine injection sites.

Subjects may exhibit general signs and symptoms associated with administration of a vaccine injection, including fever, chills, rash, aches and pains, nausea, headache, dizziness and fatigue. These side effects will be monitored, but are generally short term, mild to moderate severity and usually do not require treatment.

In previous VRC DNA vaccine studies, placebo and vaccine recipients were noted to have occasional asymptomatic and self-limited changes in laboratory tests such as temporary drop in white blood cell count. Urticaria has been reported as an adverse event possibly related to DNA vaccines.

Investigational DNA vaccines administered via Biojector have been associated with mild skin lesions (0.5-1.0 cm diameter) at the vaccination site. In these cases a small scab formed within 1-2 weeks after immunization and came off after a few days. The skin healed without treatment within a few weeks. One skin biopsy was obtained on Day 6 post vaccination. It showed subcutaneous and dermal perivascular lymphocytic inflammation. There were rare eosinophils and rare giant cells noted, and the infiltrate was composed entirely of CD3 positive cells. It included both $CD4^+$ and $CD8^+$ T cells. The process appears to be primarily a subcutaneous inflammatory response to vaccination with cutaneous manifestations.

There may be other unknown side effects.

8.5.3 Other Risks

Blood drawing may cause pain, bruising, fainting, and, rarely, infection at the site where the blood is taken.

Certain adverse events during apheresis procedures may be expected, such as vasovagal episodes (lightheadedness, dizziness, syncope, nausea, vomiting) related to needle insertion, and cutaneous paresthesias, chills, nausea, and heartburn caused by the citrate anticoagulant used

during the procedure. Hematoma formation and transient cutaneous neurological complaints related to needle insertion might also be seen. These events occur in 1-2% of donations from healthy volunteer blood donors. Vasovagal reactions are handled by postural manipulation and fluid administration. Citrate reactions are usually relieved by slowing the rate of the anticoagulant infusion and by administering oral calcium carbonate tablets (such as Tums®). Rarely, machine malfunction may results in loss of as much as a single unit (500 mL) of whole blood.

We do not know the possible effects of the study vaccines on the fetus or nursing infant. Women of reproductive potential will be required to agree to use birth control for sexual intercourse beginning 21 days prior to enrollment and continue through 4 weeks after the last study injection. Because this is a research study, women of reproductive potential will be asked to notify the site immediately upon learning of a pregnancy during this study and will be tested for pregnancy prior to administration of each study injection. The amount of blood drawn will be reduced. The subject will be contacted to ask about the outcome of a pregnancy that begins during the study.

It is possible that the standard medical tests performed as part of this research protocol will result in new diagnoses. Depending upon the medical findings and consequences of being provided with the new medical information about health status, the study subject may view this aspect of study participation as either a risk or a benefit. Any such information will be shared and discussed with the subject and, if requested by the subject, will be forwarded to the subject's primary health care provider for further workup and management.

8.5.4 Study Benefits

Study subjects will not receive direct health benefit from study participation.

This protocol is not designed to provide treatment for any condition. Others may benefit from knowledge gained in this study that may aid in the development of an avian influenza virus vaccine. The investigational DNA vaccine or investigational MIV are not expected to provide protection from influenza. Receiving the DNA vaccine may change the overall response to the licensed TIV vaccines.

8.6 PLAN FOR USE AND STORAGE OF BIOLOGICAL SAMPLES

The plan for use and storage of biological samples from this protocol is as outlined in the following sections.

8.6.1 Use of Samples, Specimens and Data

Samples, specimens and data collected under this protocol may be used to conduct protocol-related safety and immune response evaluations, exploratory laboratory evaluations related to the type of infection the study agent was designed to prevent, exploratory laboratory evaluations related to vaccine or infectious disease research in general and for research assay validation. Genetic testing may be performed in accordance with the genetic testing information that was included in the study informed consent.

8.6.2 Storage and Tracking of Blood Samples and Other Specimens

All of the stored study research samples are labeled by a code (such as a number) that only the VRC Clinic can link to the subject. Samples are stored at the NIAID Vaccine Immune T-Cell and Antibody Laboratory (NVITAL), Gaithersburg, MD or VRC Laboratories in Building 40,

which are both secure facilities with limited access. Data will be kept in password-protected computers. Only investigators or their designees will have access to the samples and data. Samples will be tracked in the Laboratory Information Management System (LIMS) database or using another software designed for this purpose (e.g., Freezerworks).

8.6.3 Disposition of Samples, Specimens and Data at Completion of the Protocol

In the future, other investigators (both at NIH and outside) may wish to study these samples and/or data. IRB approval must be sought prior to any sharing of samples. Any clinical information shared about those samples would similarly require prior IRB approval. The research use of stored, unlinked or unidentified samples may be exempt from the need for prospective IRB review and approval. Exemption requests will be submitted in writing to the NIH Office of Human Subjects Research, which is authorized to determine whether a research activity is exempt.

At the time of protocol termination, samples will remain in the NVITAL facility or VRC laboratories or, after IRB approval, transferred to another repository. Regulatory oversight of the stored samples and data may be transferred to a stored samples protocol as part of the IRB-approved termination plan. Data will be archived by the VRC in compliance with requirements for retention of research records, or after the IRB and the IND sponsor approval, it may be either destroyed or transferred to another repository.

8.6.4 Loss or Destruction of Samples, Specimens or Data

The NIH Intramural Protocol Deviation definition related to loss of or destruction of samples or data will be followed. Any loss or unanticipated destruction of samples (for example, due to freezer malfunction) or data (for example, misplacing a printout of data with identifiers) that compromises the scientific integrity of the study will be reported to the IRB in accordance with institutional policies. The PI will also notify the IRB if the decision is made to destroy the remaining samples.

8.7 SUBJECT IDENTIFICATION AND ENROLLMENT OF STUDY PARTICIPANTS

All study activities will be carried out at the Clinical Center at the NIH. Study subjects will be recruited through on-site and off-site advertising done for the screening protocol, VRC 500 (11-I-0164). Effort will be made to include women and minorities in proportions similar to that of the community from which they are recruited. Because this phase I study is designed to establish safety of the vaccines in healthy adults, enrollment will be limited to persons at least 18 years of age, and no older than 60 years of age.

8.7.1 Participation of Children

Children are not eligible to participate in this clinical trial because the investigational vaccine regimens have not been previously evaluated in adults. If the regimens are assessed as safe and immunogenic, other protocols designed for children may be conducted in the future.

8.7.2 Participation of NIH Employees

NIH employees and members of their immediate families may participate in this protocol. We will follow the Guidelines for the Inclusion of Employees in NIH Research Studies and will give each employee a copy of the “NIH Information Sheet on Employee Research Participation.” Neither participation nor refusal to participate will have an effect, either beneficial or adverse, on

the participant's employment or work situation. The NIH information sheet regarding NIH employee research participation will be distributed to all potential subjects who are NIH employees. The employee subject's privacy and confidentiality will be preserved in accordance with NIH Clinical Center and NIAID policies. For NIH employee subjects, consent will be obtained by an individual who is independent of the employee's team. If the individual obtaining consent is a co-worker to the subject, independent monitoring of the consent process will be included through the Bioethics Consultation Service. Protocol study staff will be trained on obtaining potentially sensitive and private information from co-workers or subordinates.

8.8 COMPENSATION

Subjects will be compensated for time and inconvenience in accordance with the standards for compensation of the Clinical Research Volunteer Program. The compensation per visit will be $275 for visits that include injections, and $175 for clinic visits which include a blood draw. Compensation for any clinic visit that does not include a blood draw or procedure will be $75, and compensation for completion of electronic diary card will be $25. Compensation will be $375 if apheresis is performed. The total compensation for the subject is based on the number of study clinic visits, injections completed and if optional research blood collections are performed. The approximate total compensation is $1650 without apheresis and $2025 with apheresis.

8.9 SAFETY MONITORING, PROTOCOL SAFETY REVIEW TEAM

Close cooperation between the designated members of the Protocol Team will occur to evaluate and respond to individual adverse events in a timely manner. A VRC Safety Officer (nurse practitioner or physician) conducts a daily safety review of clinical data per VRC Clinic practice. The Protocol Safety Review Team (PSRT) includes designated team members (Principal Investigator, Associate Investigators, Study Coordinator, Protocol Specialist, and other Study Clinicians). The PSRT will review the summary study safety data reports on a weekly basis through 4 weeks after the last subject receives the final study injection in order to be certain that the vaccine has an acceptable safety profile and will continue to monitor the study safety data reports on at least a monthly basis through completion of the study.

9. ADMINISTRATIVE AND LEGAL OBLIGATIONS

9.1 PROTOCOL AMENDMENTS AND STUDY TERMINATION

Protocol amendments must be made only with the prior approval of the IND sponsor, VRC, NIAID. Agreement from the investigator must be obtained for all protocol amendments and amendments to the informed consent document. All study amendments will be submitted to the IRB for approval.

The VRC, the Principal Investigator, the NIAID IRB, NIAID and the FDA reserve the right to terminate the study. The investigator will notify the IRB in writing of the study's completion or early termination.

9.2 STUDY DOCUMENTATION AND STORAGE

The investigator will maintain a list of appropriately qualified persons to whom trial duties have been delegated.

Source documents are original documents, data, and records from which the subject's data are obtained. These include but are not limited to hospital records, clinical and office charts,

laboratory and pharmacy records, diaries, microfiches, radiographs, and correspondence.

The investigator and staff are responsible for maintaining a comprehensive and centralized filing system of all study-related (essential) documentation, suitable for inspection at any time by representatives from the VRC, IRB, FDA, and/or applicable regulatory authorities. Elements include:

- Subject files containing completed informed consent forms, and supporting copies of source documentation (if kept)
- Study files containing the protocol with all amendments, Investigator Brochures, and copies of all correspondence with the IRB and VRC

In addition, all original source documentation must be maintained and be readily available.

All essential documentation should be retained by the institution for the same period of time required for medical records retention. The FDA requires study records to be retained for two years after marketing approval or refusal (21 CFR 312.62). No study document should be destroyed without prior written agreement between the VRC, and the Principal Investigator. Should the investigator wish to assign the study records to another party or move them to another location, VRC must be notified in writing of the new responsible person and/or the new location.

9.3 DATA COLLECTION AND PROTOCOL MONITORING

9.3.1 Data Collection

Clinical research data will be collected in a secure electronic web-based clinical data management system (CDMS) through a contract research organization, EMMES (Rockville, MD). Extracted data without patient identifiers will be sent to the Protocol Statistician for statistical analysis.

9.3.2 Source Documents

The site will maintain appropriate medical and research records for this trial, in compliance with ICH-GCP, regulatory and institutional requirements for the protection of confidentiality of subjects.

Source data are all information, original records of clinical findings, observations, or other activities in a clinical trial necessary for the reconstruction and evaluation of the trial. Examples of these original documents and data records include, but are not limited to, medical records, laboratory reports, pharmacy records and other research records maintained for the clinical trial.

9.3.3 Protocol Monitoring Plan

The NIAID and VRC or their authorized representatives are responsible for contacting and visiting the investigator for the purpose of inspecting the facilities and, upon request, inspecting the various records of the trial, provided that subject confidentiality is respected.

Site investigators will allow the study monitors, the NIAID IRB, and the FDA to inspect study documents (*e.g.*, consent forms, drug distribution forms, and case report forms) and pertinent hospital or clinic records for confirmation of the study data.

Site visits by study monitors will be made in accordance with the study monitoring plan to monitor the following: study operations, the quality of data collected in the research records, the

accuracy and timeliness of data entered in the database, and to determine that all process and regulatory requirements are met. Study monitoring visits will occur at initiation of the study, at intervals determined by the IND Sponsor during conduct of the study and at completion of the study.

9.4 LANGUAGE

All written information and other material to be used by subjects and investigative staff must use vocabulary and language that are clearly understood.

9.5 POLICY REGARDING RESEARCH-RELATED INJURIES

The Clinical Center will provide short-term medical care for any injury resulting from participation in this research. In general, the NIH, the Clinical Center, or the Federal Government will provide no long-term medical care or financial compensation for research-related injuries.

Serious injuries related to the H7N9 MIV may be covered by the Public Readiness and Emergency Preparedness (PREP) Act. Information about this program can be found at this website: http://www.hrsa.gov/cicp/ .

10. REFERENCES

1. Subbarao, K., B.R. Murphy, and A.S. Fauci, *Development of effective vaccines against pandemic influenza.* Immunity, 2006. **24**(1): p. 5-9.
2. CDC. *Types of Influenza Viruses*. 2014 4/15/2014]; Available from: http://www.cdc.gov/flu/about/viruses/types.htm.
3. WHO, *Influenza (Seasonal) Fact Sheet*. 2009: Geneva, Switzerland.
4. Kilbourne, E., *Influenza pandemics of the 20th century.* Emerg Infect Dis [serial on the Internet]. 2006. **available from http://www.cdc.gov/ncidod/EID/vol12no01/05-1254.htm**.
5. WHO. *Human infections with avian influenza A(H7N9) virus*. 2014; Available from: http://www.who.int/influenza/human_animal_interface/influenza_h7n9/Risk_Assessment/en/.
6. CDC. *Avian Influenza A (H7N9) Virus*. 2014 4/15/2014]; Available from: http://www.cdc.gov/flu/avianflu/h7n9-virus.htm.
7. Abdelwhab, E.M., J. Veits, and T.C. Mettenleiter, *Prevalence and control of H7 avian influenza viruses in birds and humans.* Epidemiol Infect, 2014. **142**(5): p. 896-920.
8. Jones, J.C., et al., *Possible Role of Songbirds and Parakeets in Transmission of Influenza A(H7N9) Virus to Humans*. Emerg Infect Dis, 2014. **20**(3).
9. Fiore, A.E., et al., *Prevention and control of influenza: recommendations of the Advisory Committee on Immunization Practices (ACIP), 2008.* MMWR Recomm Rep, 2008. **57**(RR-7): p. 1-60.
10. Harper, S.A., et al., *Prevention and control of influenza: recommendations of the Advisory Committee on Immunization Practices (ACIP).* MMWR Recomm Rep, 2004. **53**(RR-6): p. 1-40.
11. Targonski, P.V., R.M. Jacobson, and G.A. Poland, *Immunosenescence: role and measurement in influenza vaccine response among the elderly*. Vaccine, 2007. **25**(16): p. 3066-9.
12. Ambrozaitis, A., et al., *A novel mammalian cell-culture technique for consistent production of a well-tolerated and immunogenic trivalent subunit influenza vaccine.* Vaccine, 2009. **27**(43): p. 6022-9.
13. Treanor, J.J., et al., *Protective efficacy of a trivalent recombinant hemagglutinin protein vaccine (FluBlok(R)) against influenza in healthy adults: a randomized, placebo-controlled trial.* Vaccine, 2011. **29**(44): p. 7733-9.
14. Yan, J., et al., *Protective immunity to H7N9 influenza viruses elicited by synthetic DNA vaccine.* Vaccine, 2014. **32**(24): p. 2833-42
15. Goff, P.H., et al., *Induction of cross-reactive antibodies to novel H7N9 influenza virus by recombinant Newcastle disease virus expressing a North American lineage H7 subtype hemagglutinin.* J Virol, 2013. **87**(14): p. 8235-40.
16. Smith, G.E., et al., *Development of influenza H7N9 virus like particle (VLP) vaccine: homologous A/Anhui/1/2013 (H7N9) protection and heterologous A/chicken/Jalisco/CPA1/2012 (H7N3) cross-protection in vaccinated mice challenged with H7N9 virus.* Vaccine, 2013. **31**(40): p. 4305-13.
17. Ledgerwood, J.E. and B.S. Graham, *DNA vaccines: a safe and efficient platform technology for responding to emerging infectious diseases*. Hum Vaccin, 2009. **5**(9): p. 623-6.

18. Wei, C.J., et al., *Induction of broadly neutralizing H1N1 influenza antibodies by vaccination.* Science, 2010. **329**(5995): p. 1060-4.
19. Wei, C.J., et al., *Elicitation of broadly neutralizing influenza antibodies in animals with previous influenza exposure.* Science Translational Medicine, 2012. **4**(147): p. 147ra114.
20. Ledgerwood, J.E., et al., *Influenza virus H5 DNA vaccination is immunogenic by intramuscular and intradermal routes in humans.* Clinical and Vaccine Immunology, 2012. **19**(11): p. 1792-7.
21. Ledgerwood, J.E., et al., *DNA priming and influenza vaccine immunogenicity: two phase 1 open label randomised clinical trials.* Lancet Infect Dis, 2011. **11**(12): p. 916-924.
22. Ledgerwood, J.E., et al., *Prime-Boost Interval Matters: A Randomized Phase 1 Study to Identify the Minimum Interval Necessary to Observe the H5 DNA Influenza Vaccine Priming Effect.* J Infect Dis, 2013. **208**(3): p. 418-22.
23. Khurana, S., et al., *DNA Priming Prior to Inactivated Influenza A(H5N1) Vaccination Expands the Antibody Epitope Repertoire and Increases Affinity Maturation in a Boost-Interval-Dependent Manner in Adults.* J Infect Dis, 2013. **208**(3): p. 413-7.
24. Wang, S., et al., *Heterologous HA DNA vaccine prime-inactivated influenza vaccine boost is more effective than using DNA or inactivated vaccine alone in eliciting antibody responses against H1 or H3 serotype influenza viruses.* Vaccine, 2008. **26**(29-30): p. 3626-33.
25. Gao, R., et al., *Human infection with a novel avian-origin influenza A (H7N9) virus.* N Engl J Med, 2013. **368**(20): p. 1888-97.
26. Tavel, J.A., et al., *Safety and immunogenicity of a Gag-Pol candidate HIV-1 DNA vaccine administered by a needle-free device in HIV-1-seronegative subjects.* J Acquir Immune Defic Syndr, 2007. **44**(5): p. 601-5.
27. Graham, B.S., et al., *Phase 1 safety and immunogenicity evaluation of a multiclade HIV-1 DNA candidate vaccine.* J Infect Dis, 2006. **194**(12): p. 1650-1660.
28. Catanzaro, A.T., et al., *Phase I clinical evaluation of a six-plasmid multiclade HIV-1 DNA candidate vaccine.* Vaccine, 2007. **25**(20): p. 4085-92.
29. Koup, R.A., et al., *Priming Immunization with DNA Augments Immunogenicity of Recombinant Adenoviral Vectors for Both HIV-1 Specific Antibody and T-Cell Responses.* PLoS One, 2010. **5**(2): p. e9015.
30. Kibuuka, H., et al., *A phase 1/2 study of a multiclade HIV-1 DNA plasmid prime and recombinant adenovirus serotype 5 boost vaccine in HIV-Uninfected East Africans (RV 172).* J Infect Dis, 2010. **201**(4): p. 600-7.
31. Jaoko, W., et al., *Safety and immunogenicity study of Multiclade HIV-1 adenoviral vector vaccine alone or as boost following a multiclade HIV-1 DNA vaccine in Africa.* PLoS One, 2010. **5**(9): p. e12873.
32. Martin, J.E., et al., *A DNA vaccine for Ebola virus is safe and immunogenic in a phase I clinical trial.* Clin Vaccine Immunol, 2006. **13**(11): p. 1267-77.
33. Martin, J.E., et al., *A SARS DNA vaccine induces neutralizing antibody and cellular immune responses in healthy adults in a Phase I clinical trial.* Vaccine, 2008. **26**(50): p. 6338-43.
34. Martin, J.E., et al., *A West Nile virus DNA vaccine induces neutralizing antibody in healthy adults during a phase 1 clinical trial.* J Infect Dis, 2007. **196**(12): p. 1732-40.

35. Ledgerwood, J.E., et al., *A West Nile virus DNA vaccine utilizing a modified promoter induces neutralizing antibody in younger and older healthy adults in a phase I clinical trial.* J Infect Dis, 2011. **203**(10): p. 1396-404.
36. Graham, B.S., et al. *Evaluation of candidate DNA HIV-1 vaccine delivery by Biojector or needle and syringe in healthy adults (VRC 008)*. in *AIDS Vaccine 2007*. 2007. Seattle, WA.
37. Couch, R.B., et al., *A randomized clinical trial of an inactivated avian influenza A (H7N7) vaccine.* PLoS One, 2012. **7**(12): p. e49704.
38. Cox, R.J., et al., *A phase I clinical trial of a PER.C6 cell grown influenza H7 virus vaccine.* Vaccine, 2009. **27**(13): p. 1889-97.
39. WHO. *Candidate vaccine viruses for avian influenza A(H7N9)*. 2014; Available from: http://www.who.int/influenza/vaccines/virus/candidates_reagents/a_h7n9/en/.
40. Bart, S.A., et al., *A Cell Culture-Derived MF59-Adjuvanted Pandemic A/H7N9 Vaccine Is Immunogenic in Adults.* Sci Transl Med, 2014. **6**(234): p. 234ra55.
41. Betts, M.R., J.P. Casazza, and R.A. Koup, *Monitoring HIV-specific CD8+ T cell responses by intracellular cytokine production.* Immunol Lett, 2001. **79**(1-2): p. 117-25.
42. Helms, T., et al., *Direct visualization of cytokine-producing recall antigen-specific CD4 memory T cells in healthy individuals and HIV patients.* J Immunol, 2000. **164**(7): p. 3723-32.
43. Barouch, D.H., et al., *A human T-cell leukemia virus type 1 regulatory element enhances the immunogenicity of human immunodeficiency virus type 1 DNA vaccines in mice and nonhuman primates.* J Virol, 2005. **79**(14): p. 8828-34.
44. Yang, Z.Y., et al., *A DNA vaccine induces SARS coronavirus neutralization and protective immunity in mice.* Nature, 2004. **428**(6982): p. 561-4.

APPENDIX I
Informed Consent Form

Text to be included in the NIH Clinical Center Informed Consent Template

TITLE: VRC 315: A Phase I Open-Label, Randomized Study of H7 Influenza Prime-Boost Regimens in Healthy Adults: Recombinant H7 DNA Plasmid Vaccine, VRC-FLUDNA071-00-VP, Administered Alone or with Monovalent Influenza Subunit Virion H7N9 Vaccine (MIV) as Prime with MIV Boost Compared to MIV Prime with MIV Boost

INTRODUCTION

We invite you to take part in a research study at the National Institutes of Health (NIH).

First, we want you to know that:

> Taking part in NIH research is entirely voluntary.
>
> You may choose not to take part, or you may withdraw from the study at any time. In either case, you will not lose any benefits to which you are otherwise entitled. However, to receive care at the NIH, you must be taking part in a study or be under evaluation for study participation.
>
> You may receive no benefit from taking part. The research may give us knowledge that may help people in the future.

Second, some people have personal, religious or ethical beliefs that may limit the kinds of medical or research treatments they would want to receive (such as blood transfusions). If you have such beliefs, please discuss them with your NIH doctors or research team before you agree to the study.

Now we will describe this research study. Before you decide to take part, please take as much time as you need to ask any questions and discuss this study with anyone at NIH, or with family, friends or your personal physician or other health professional.

PURPOSE OF THE STUDY

This is a study of two experimental vaccines for prevention of avian influenza ("bird flu"). "Experimental" means that the study vaccines have not been approved by the Food and Drug Administration (FDA) for preventing of avian influenza infection. The FDA allows their use in research only. It is not known if the vaccines work. The plan is to enroll about 30 people in this study. The main purpose of this study is to see if the experimental vaccines are safe and whether there are any side effects. We also want to study immune responses to these vaccines.

You are eligible to participate in this study because you have completed the screening process, completed the assessment of understanding, are between 18 and 60 years old, have laboratory test results that meet eligibility requirements and you do not have any significant medical problems as determined during your participation in the screening protocol.

The study will be done by the Vaccine Research Center (VRC) with study visits occurring at the NIH Clinical Center in Bethesda, MD. Study participation will last about 28 weeks. While on the study, you will be monitored for vaccine-related side effects. In this study, the experimental

vaccines will be given to people by injections (shots) in the upper arms.

You will be told of any new information learned during this study that might cause you to change your mind about staying in the study. At the end of the study, you will be told when study results may be available and how to learn about them.

STUDY VACCINES

Vaccines are used to try to create resistance (or immunity) to prevent an infection. The vaccines in this study are intended to help the body to develop an immune response to avian influenza type H7N9. In this consent the two vaccines are called "H7 DNA Vaccine (DNA vaccine)" and "H7N9 Monovalent Inactivated Vaccine (MIV)". It is not known if the study vaccines work to protect against avian influenza. You cannot get an avian flu infection from either study vaccine. Neither of the study vaccines contain avian influenza virus.

DNA Vaccine: Most vaccines are made of proteins and injected into a muscle. Proteins are natural substances that the body uses as building blocks. DNA serves as nature's code (instructions) for protein production in the body. A new kind of vaccine being tested in this study is made from the DNA that is the code for an avian influenza protein. In this study, the DNA will be injected into a muscle. It will instruct the body to make a small amount of the influenza protein. The DNA vaccine was developed by the NIH. The DNA vaccine that will be given in this study has not been tested in people. However, similar vaccines for seasonal influenza and for other avian flu influenza were tested in many people. There were no severe side effects related to these vaccines.

MIV: The MIV for this study was made by a method similar to that used to make the licensed seasonal influenza vaccine offered every year. The MIV was developed by Sanofi-Pasteur, a company that specializes in making vaccines. The MIV vaccine in this study has been given in other studies to about 200 people. Safety follow-up for subjects enrolled in these studies is still ongoing.

Studies of a similar inactivated avian flu H5N1 vaccine were conducted in many people. There were no severe side effects.

Studies conducted for H5N1 avian flu vaccines so far suggest that H5 DNA vaccination followed by inactivated H5N1 MIV vaccination gives a better immune response than 2 injections of either vaccine alone. This study will look at immune responses to the two influenza vaccines targeted to H7N9 avian flu more closely to see if the findings can be reproduced for H7N9 influenza.

STUDY PROCEDURES

If you agree to take part in the study you will be randomly assigned (like pulling a number from a hat) to one of 3 groups. All injections will be given in the upper arm muscle. This is called an intramuscular "IM" injection. Neither you nor the clinic staff will be informed about what type of vaccine you are receiving until after you are enrolled. Your schedule will be learned on the day of enrollment. The 3 possible injection schedules are shown in the following table:

Protocol VRC 315		Vaccination Schedules for each Group	
**Group**	**Subjects***	**Day 0**	**Week 16**
Group 1	10	H7 DNA 4 mg	H7N9 MIV
Group 2	10	H7 DNA + H7N9 MIV	H7N9 MIV
Group 3	10	H7N9 MIV	H7N9 MIV
TOTAL	30	All injections given in upper arm muscle,<br>On Day 0 in Group 2, 2 injections will be administered in different arms.	
*Up to 2 subjects over target are allowed per group			

You will have 2 telephone follow-up contacts and about 8 clinic visits during this study. Studies of investigational vaccines require following a set schedule for injections and follow-up visits in order to answer the study research questions. Some flexibility in scheduling is permitted, but it is important that you work with the staff to stay on schedule. Study visits may take 2 to 4 hours to complete.

You will receive vaccinations at two different times during the study. The day of your first vaccination is called Day 0. Depending on the study group, on Day 0 you may get one or two injections. There will be 16 weeks between vaccinations. Everyone will receive one injection at the second vaccination.

The inactivated H7N9 vaccine injections will be given using a needle and syringe. DNA vaccine injections will be given using a needleless system called the Biojector 2000®. This device delivers the vaccine through the skin without the use of a needle. It uses the pressure of carbon dioxide, instead of a needle, to inject the vaccine through your skin and into the muscle. Needleless systems have been used since 1947 to deliver vaccines and other types of drugs. This system has FDA clearance for delivering vaccine injections into muscles.

The clinic staff will observe you for at least 30 minutes after each vaccination. One to two days after each study injection the clinic staff will contact you to check on how you are doing. You will be asked to complete a diary card at home during the week after injections. This will require that you record your temperature and symptoms and look at the injection site each day. You will be provided with a thermometer to take your temperature and ruler to measure any injection site skin changes. You will be asked to record any symptoms and report any side effects to one of the study physicians or nurses as soon as possible. The clinic staff is available to you by phone 24 hours per day.

You may record your symptoms on a paper diary card or enter them into a secure electronic form using the internet. If you choose to report your symptoms through the internet you will be trained by the clinic staff and given a username and password, and you will be compensated for your time and inconvenience for completion of a diary card over the internet. If you have any symptoms, it may be necessary to come to the study clinic for an examination before your next scheduled visit. It is very important that you follow the instructions given to you by the clinic staff.

At each visit, you will be checked for any health changes or problems since your last visit. You will be asked how you are feeling and if you have taken any medications. Blood will be drawn at scheduled study visits. Urine samples may be collected if needed to check on your health. You

will be told promptly if any of your test results show a health problem. Some blood samples will be used to study your immune response to the vaccine. Results of immune response tests are not used to check on your health and will not be given to you during the study.

The amount of blood drawn will be within the guidelines of the NIH Clinical Center and will vary from about 13 tubes (~ 8 tablespoons) to about 23 tubes (~ 14 tablespoons), depending on the visit. We expect about 7 visits, but you may also be asked to have laboratory tests or an exam between regular visits if needed to evaluate a change in your health.

If you have serious side effects, the study physician may decide that you should not receive any further injections. However, you will be asked to continue your study follow-up visits even if you do not get the full set of injections. You will be asked to continue to have blood tests that monitor your health and to complete the blood drawing done for research lab tests.

Apheresis: About 2-3 weeks after your second study injection we would like to collect your blood sample by a method called "apheresis." In this procedure, blood will be removed through a needle in an arm, spun in a machine that separates the desired blood component (white blood cells or plasma), and the remainder will be returned to you. Citrate, a medication to prevent blood from clotting, will be added to the blood while in the machine to prevent it from clotting. This procedure will be done at the Department of Transfusion Medicine in the NIH Clinical Center.

The purpose of the apheresis in this study is to allow the investigator to obtain a larger number of white blood cells than can be collected by simple blood drawing. More white blood cells will allow more laboratory tests to be done to see how the immune system responds to the study vaccine. The number of white blood cells collected is a small fraction of the total amount in your body. The body quickly replaces removed cells. Similar procedures are used on a daily basis in Blood Banks. The procedure will take approximately 45-90 minutes.

Before apheresis is done, your weight, pulse and blood pressure will be checked. You will be asked questions about your general health and medical history. You will be asked to sign the Department of Transfusion Medicine's consent form for the apheresis procedure on the day of apheresis. You will be asked to lie on a recliner or couch. The kits used to collect the apheresis products are sterilized, single-use, disposable sets that are not in contact with any person's body fluids other than yours. No blood products are given to you during these procedures. A physician from the Department of Transfusion Medicine will be available in or near the apheresis donor area at all times.

MONITORING OF THE STUDY

This study will be monitored by a group of physicians and scientists at the NIH Clinical Center. This group will review the information from the study and will pay close attention to harmful reactions. If it is decided that significant reactions have occurred, further injections may be delayed or canceled.

GENETIC TESTING

Some of the blood drawn from you as part of this study will be used for genetic tests. In research studies some genetic tests are done to see if different types of immune response seem to be related to genetic differences in people. Genetic tests done in a research lab from your stored

samples will not be recorded in your medical record and will not have your name on the sample. The performance of these tests is not for health care purposes, and you will not be notified of these results.

Human Leukocyte Antigen (HLA) type is a genetic test that may be ordered through the NIH Clinical Center medical laboratory. If performed at the NIH Clinical Center, your HLA type results will be in your medical record. People with certain HLA types might be more likely to develop certain diseases. Simply having those HLA types doesn't mean they will develop those diseases. It is our policy not to discuss your HLA results unless they have direct medical or reproductive implications for you or your family. Genetic information about you will not be revealed to others, including your relatives, without your permission. We will not release any information about you or your family to any insurance company or employer unless you sign a document allowing release of information.

STORED SAMPLES

During your participation on this study blood samples will be collected from you. We will store these samples for future research to learn more about influenza virus, vaccines, the immune system, and/or other medical conditions. The results from the research done with your stored samples are not meant to be used for medical care. They will not be given to your health care provider and will not be put in your medical record.

Labeling of Stored Samples

Your stored samples will be labeled by a code (such as a number) that only the study team can link to you. Any identifying information about you will be kept confidential to the extent permitted by law.

Risks from Stored Samples

The greatest risk is the unplanned release of information from your medical records. The chance that this information will be given to an unauthorized person without your permission is very small. Possible problems with the unplanned release of information include discrimination when applying for insurance and employment. Similar problems may occur if you disclose information yourself or agree to have your medical records released.

Future Studies

In the future, other investigators at NIH or outside of NIH may wish to study your stored samples. When the study team shares your stored samples, they will be marked with a code, but will not have any identifying information on them. Some information about you, such as your gender, age, health history, or ethnicity may also be shared with other investigators. Any future research studies using your samples will be reviewed by the investigator's Institutional Review Board (IRB), a special committee that oversees medical research studies to protect the rights and welfare of human subjects.

Your stored materials will be used only for research and will not be sold. The research done with your materials may be used to develop new products in the future but you will not receive payment for such products.

Making your Choice: Refusal to let us collect or store your blood samples means that you are not eligible to participate in this study. If you agree to participate in this study, this means you

also agree to let us indefinitely store any of your samples for future research. Your decision not to participate does not affect your eligibility for other studies at NIH.

POSSIBLE STUDY RISKS

***Possible* risks from the injections:** temporary stinging, pain, redness, soreness, itchiness, swelling, bruising, or a cut in the arm. There is a very small chance of infection.

***Possible* risks of blood drawing:** pain, bleeding, bruising, feeling lightheaded, fainting, or rarely, infection at the site where the blood is taken.

***Possible* risks of apheresis:** apheresis donations are generally safe and side effects are rare. Pain, bruising or discomfort at the needle placement site may occur. Sometimes apheresis causes a tingling sensation around the mouth or in the finger, chills, nausea, heartburn or mild muscle cramps. This can usually be relieved by slowing or temporarily interrupting the apheresis or taking a calcium containing antacid, such as Tums®. Other possible side effects are anxiety, vomiting and lightheadedness. Temporary lowering of the blood pressure may develop. There is the rare possibility of infection, fainting or seizure. Very rarely a nerve problem at the needle placement site may occur. Also, very rarely, a machine malfunction may occur, resulting in the loss of about one unit (one pint) of blood.

There are theoretical risks from re-infusion of the blood after processing by the machine such as infection or an adverse reaction to the blood components. However, this has not been seen in many thousands of volunteers who have undergone this or similar procedures to date. There may be additional risks of apheresis that are unknown at this time.

***Possible* risks from genetic testing:** unintended release of information that could be used by insurers or employers; discovering a gene or HLA type that suggests risk of disease for you or your family; discovering undisclosed family relationships.

***Possible* risks from any vaccine:** fever, chills, rash, aches and pains, nausea, headache, dizziness, and fatigue. Some people have allergic reactions to vaccines. These types of reactions are usually greatest within the first 24 hours after vaccination and typically last 1 to 3 days. Over-the-counter medicine, such as acetaminophen, will generally help relieve symptoms from vaccination and may be used.

***Possible* risks from DNA vaccine:** temporary drop in white blood cell count, sore arm, skin rash or hives, some people get a small red bump and then a scab for a few days where the shot is given. No safety studies in animals have been done for the H7 DNA vaccine. Experience so far in more than a decade of testing with other DNA vaccines made by the VRC has been that the vaccinations were safe and well-tolerated.

***Possible* risks from MIV:** fever, muscle and general body aches, headache, fatigue, hoarseness, sore, red or itchy eyes, cough, itching, and nausea. A severe allergic reaction can occur especially in people that are allergic to eggs. In 1976, a small number of people who got an inactivated swine flu vaccine developed a severe nerve weakness called Guillain-Barré

syndrome. This has not been seen consistently with other influenza vaccines. Most persons who develop Guillain-Barré recover. Four days after vaccination with MIV in a clinical trial, a 52-year-old man had a "heart attack" (myocardial infarction) from which he recovered. An association with vaccination could not be ruled out. The risks discussed are based on experience with other similar inactivated flu vaccines.

Serious injuries related to the MIV may be covered by the Public Readiness and Emergency Preparedness (PREP) Act. Information about this program can be found at this website: http://www.hrsa.gov/cicp/ . This program does not apply to the DNA vaccine.

***Unknown* safety risks:** There may be side effects from the study vaccines - even serious or life threatening ones- that we do not yet know about. Please tell the study staff about any side effect you think you are having. This is important for your safety.

***Possible* risks from Pregnancy:** If you are pregnant, breast-feeding or want to become pregnant during the next 20 weeks, you cannot participate. We do not know the possible effects of the study vaccines on the fetus or nursing infant. Therefore, women who are able to become pregnant must have a negative pregnancy test before each immunization and agree to practice adequate birth control beginning at least 21 days prior to receiving the first injection until 4 weeks after the last injection. Adequate methods of birth control include: condoms, male or female, with or without a spermicide; diaphragm or cervical cap with spermicide; intrauterine device; all prescription methods (such as contraceptive pills, injections, patches and others); or a male partner who has previously undergone a vasectomy. You must notify the clinic staff immediately upon learning that you have become pregnant during this study. You must also notify the clinic if you suspect that you **might** be pregnant during this study. If you become pregnant, you will receive no further injections. However, you will be asked to continue with the planned study follow-up visits. You will be asked about the outcome of the pregnancy.

***Other* Risks:** It is unknown if the study vaccine may alter your response to any future infections you may have with influenza viruses.

You will be made aware of significant health effects of the vaccine and serious side effects if they occur in other participants. You will be updated during the trial as necessary.

You may not donate blood at a blood bank while participating in this research study or for one year after the date of the last experimental vaccine injection.

POSSIBLE BENEFITS

This study is not designed to benefit you. It is unknown if the vaccines will work. The study is not designed to protect participants from avian flu. You and others may benefit in the future from the information that will be learned from the study.

COSTS TO YOU FOR YOUR PARTICIPATION

The purpose of all of your health evaluations in this VRC clinical trial at the NIH Clinical Center is for research, not to provide health care. There are no costs to you for participating in this study.

The NIH will not charge you or your insurance carrier for any health evaluations or services provided at the NIH Clinical Center. You (or your insurance carrier) will be responsible for the costs of all medical care that you receive somewhere other than the NIH Clinical Center.

Participants will receive compensation consistent with NIAID policy to help with transportation costs and other expenses that may occur because of study participation. It is possible that you may have some expenses that are not covered by the compensation provided.

PAYMENT TO YOU FOR YOUR PARTICIPATION

You will be compensated $175 for each visit that does not include an injection but does include a blood draw and $275 for each injection visit and $375 (rather than $175) if apheresis is performed. For visits that do not include an injection or a blood draw you will be compensated $75, and compensation for completion of electronic diary card will be $25. The total compensation will be about $1650 without apheresis and $2025 with apheresis, depending on the study injections and the number of study visits you complete. You will receive compensation after each completed study visit.

REASONS FOR REMOVING YOU FROM THE STUDY WITHOUT YOUR CONSENT

You may be stopped from receiving study vaccinations for several different reasons, including:

- You don't keep appointments or follow study procedures.
- The study sponsor or study doctor decides to stop or cancel the study.
- The regulatory boards or the FDA decide that the study should be stopped.
- You get a serious illness.
- You need to receive treatment with a medication that affects your immune system (such as a steroid like prednisone).
- You have a serious side effect thought to be due to vaccination.
- You become pregnant.

If you agree to take part in this study, it is important for you to keep all your appointments. However, if you don't want to stay in the study, you can leave at any time. You will not lose any benefits that you would have had if you had not joined the study. If the second study vaccination is not given for any reason you will still be asked to continue with follow-up visits until the end of the study. It is important to continue to monitor your health even if you do not receive both study vaccinations.

ALTERNATIVES

This study is not designed to treat any disease. You may choose to not participate.

CONFLICT OF INTEREST

The National Institutes of Health reviews NIH staff researchers at least yearly for conflicts of interest. You may ask the research team for additional information or a copy of the Protocol Review Guide.

The National Institutes of Health, including some members of the Vaccine Research Center scientific staff, developed the investigational DNA vaccine being used in this research

study. The results of this study could play a role in whether the FDA will approve the vaccine for sale at some time in the future. If approved, the future sale of the vaccine could lead to payments to NIH and some NIH scientists. By U.S. law, government scientists are required to receive such payments for their inventions. You will not receive any money from the development or sale of the product.

The H7N9 MIV vaccine is made by Sanofi-Pasteur, a company that specializes in making vaccines. The vaccine is being provided to us by Sanofi-Pasteur. If approved, the future sale of the vaccine could lead to payments to Sanofi Pasteur. You will not receive any money from the development or sale of the product.

This protocol may have investigators who are not NIH employees. Non-NIH investigators are expected to adhere to the principles of the Protocol Review Guide but are not required to report their personal financial holdings to the NIH.

OTHER PERTINENT INFORMATION

1. Confidentiality. When results of an NIH research study are reported in medical journals or at scientific meetings, the people who take part are not named and identified. In most cases, the NIH will not release any information about your research involvement without your written permission. However, if you sign a release of information form, for example, for an insurance company, the NIH will give the insurance company information from your medical record. This information might affect (either favorably or unfavorably) the willingness of the insurance company to sell you insurance.

The Federal Privacy Act protects the confidentiality of your NIH medical records. However, you should know that the Act allows release of some information from your medical record without your permission, for example, if it is required by the Food and Drug Administration (FDA), members of Congress, law enforcement officials, or other authorized people such as the vaccine manufacturer and the study sponsor.

2. Policy Regarding Research-Related Injuries. The Clinical Center will provide short-term medical care for any injury resulting from your participation in research here. In general, no long-term medical care or financial compensation for research-related injuries will be provided by the National Institutes of Health, the Clinical Center, or the Federal Government. However, you have the right to pursue legal remedy if you believe that your injury justifies such action.

3. Payments. The amount of payment to research volunteers is guided by the National Institutes of Health policies.

4. Problems or Questions. If you have any problems or questions about this study or about any research-related injury, contact the Principal Investigator, Julie Ledgerwood, D.O., at 301-451-8715 or the Study Coordinator, Kathryn Zephir, R.N. at 301-451-8715 or 1-800-599-7885.

If you have any questions about your rights as a research subject, you may call the Clinical Center Patient Representative at 301-496-2626.

5. Consent Document. Please keep a copy of this document in case you want to read it again.

COMPLETE APPROPRIATE ITEM(S) BELOW:

Adult Study Participant's Consent

I have read the explanation about this study and have been given the opportunity to discuss it and to ask questions. I hereby consent to take part in this study.

________ Time

_________________________________ ________
Signature of Adult Participant/Legal Representative Date

____
Print Name

THIS CONSENT DOCUMENT HAS BEEN APPROVED FOR USE FROM XXXXXX THROUGH XXXXXX.

_________________________________ ________ _________________________________ ________
Signature of Investigator/Person Obtaining Consent Date Signature of Witness Date

_________________________________ _________________________________
Print Name Print Name

APPENDIX II

CONTACT INFORMATION

Principal Investigator:
Julie Ledgerwood, D.O., 301-594-8502
Vaccine Research Center, NIAID, NIH
Bethesda, MD 20892

Associate Investigators:
Joseph Casazza, M.D., Ph.D. 301-594-8627
Adam Dezure, M.D., 301-402-9043
Cynthia Starr Hendel, CRNP 301-402-1341
Lasonji Holman, CFNP 301-402-8641
Sarah A. Plummer, RN, MSN, NP 301-402-8640

Study Coordinators/ Research Nurses:
Kathryn Zephir, RN, BSN, MS
VRC 315 Study Coordinator
All Clinic Staff: 301-451-8715
Ingelise Gordon, RN
Pamela Costner, RN, BSN
Brenda Larkin, RN, BSN, CCRC
Floreliz Mendoza, RN
Laura Novik, RN, MA, CCRC
Jamie Saunders, RN, BSN
William Whalen, RN, BSN

VRC Medical Officer:
Barney S. Graham, M.D., Ph.D., 301-594-8468
VRC, NIAID, NIH
40 Convent Drive
Bldg 40, Room 2502
Bethesda, MD 20892-3017

Study Site:
National Institutes of Health Clinical Center
Vaccine Evaluation Clinic 5NES, Building 10
Bethesda, MD 20892

Protocol Statistician:
Zonghui Hu, Ph.D., 301-451-2434
Biostatistics Research Branch
Division of Clinical Research, NIAID, NIH

Data Management:
Vaccine Research Center, NIAID, NIH, Bethesda, MD 20892 and
EMMES Corporation (301-251-1161)
401 N Washington St, Rockville, MD 20850

Site And Data Monitoring:
Olga Vasilenko, M.S., 301-402-8646

Scientific and Laboratory Collaborators:
Vaccine Research Center, NIAID, NIH
John Mascola, M.D., 301-594-8490
Richard Koup, M.D., 301-594-8585
Robert Bailer, Ph.D., 301-594-8481
Barney Graham, M.D., Ph.D., 301-594-8468

Research Immunology Central Laboratory:
NVITAL (NIAID Vaccine Immune T-Cell and Antibody Laboratory)
9 West Watkins Mill Road, Suite 150
Gaithersburg, MD 20878

Other Influenza Testing Labs:
Terry Tumpey, Ph.D.,
CDC, Influenza Branch,
1600 Clifton Road NE
Atlanta, GA 30333

Vaccine Manufacturers:
Sanofi Pasteur, Inc, Swiftwater, PA

Vaccine Pilot Plant (VPP), VRC, NIAID
(operated by Leidos Biomedical Research, Inc.),
Frederick, MD

Pharmacy:
Judith Starling, R.Ph.,
Hope DeCederfelt, R.Ph.
Pharmaceutical Development Section
Clinical Center, Building 10/1N257
Bethesda, MD 20892, 301-496-4363

NIH Apheresis Clinic:
Cathy Cantilena, M.D., 301-451-8637
Department of Transfusion Medicine
10 Center Drive, Building 10-MSC 1184
Bethesda, MD 20892-1184

VRC Production and Regulatory Affairs:
Richard Schwartz, Ph.D., 301-594-8485
Michelle Conan-Cibotti, Ph.D., 240-292-4938
Florence Kaltovich, M.S., 301-402-2402
Judy Stein, MPH, MBA, 734-763-7753

VRC Protocol Operations:
Mary E. Enama, M.A., PA-C, 301-594-8501
Galina Yamshchikov, M.S., 301-594-1064
Nina M. Berkowitz, M.P.H., 301-451-2896
Iris Pittman, B.A., CCRP, 301-451-8543

APPENDIX III
SCHEDULE OF EVALUATIONS

		**Screen**	**VRC 315 Schedule of Evaluations**										
**Visit**		01	02	02A	02B	03	04	04A	05	06	[5]06A	07	08
**Week of Study**		-8 to 0	W 0	W1	W2	W4	W16	W17	W17	W18	W18	W20	W28
**[1]Day of Study**		-56 to 0	D 0	D3	D8	D28	D112	D113	D117	D126	D126	D140	D196
**Clinical Evaluations**	**Tube**												
VRC 500 Screening Consent		X											
VRC 315 AoU, Informed Consent			X										
Physical exam for eligibility, height/weight/vitals at screen; vital signs and targeted exam (as needed) other visits.		X	X	X		X	X		X	X		X	X
Medical history targeted to eligibility at screen; interim history for AEs other visits		X	X			X	X					X	X
[2] Study Vaccinations			V1				V2						
Begin 7-Day Diary Card			X				X						
Telephone contact; clinic visit if indicated					X			X					
Counseling on pregnancy prevention		X	X			X	X						
CBC	EDTA	3	3	3		3	3					3	
[3] Pregnancy test: urine (or serum)		X	X			X	X					X	
Creatinine and ALT	GLT	4	4	4									
[4] HLA type	EDTA						20						
**Research Immunology**													
Serum storage	SST	80	80			40	40		40	40		40	40
PBMC and plasma for storage	EDTA	120	120			80	80		[6]80	120 [or 60 if same day as apheresis]	[5]Optional apheresis	80	80
**Daily Volume (mL)**		**207**	**207**	**7**	**-**	**123**	**143**	**-**	**120**	**160**	**[6]**	**123**	**120**
**Max. Cumulative Volume (mL)**		**207**	**414**	**421**	**-**	**544**	**687**	**-**	**807**	**967**	**973**	**1096**	**1216**

[1] VRC 500: Most screening evaluations must be no more than 56 days prior to Day 0 to be used for eligibility (pregnancy test from Day 0 must be used for eligibility). If clinical assessment on Day 0 suggests significant changes may have occurred since screening, then physical examination & laboratory studies done on Day 0 are used for eligibility. Day 0=day of enrollment and first vaccine injection. Day 0 evaluations prior to first injection are the baseline for assessing adverse events subsequently.
[2] Complete post vaccination evaluations (temperature, BP, pulse, respiration, and injection site assessment) at 30 minutes or longer after each study injection.
[3] Negative pregnancy test results must be confirmed for women of reproductive potential prior to administering the vaccine injection.
[4] HLA type blood sample is collected once at any timepoint in the study and is shown as a Visit 04 evaluation for convenience; however, if HLA type is already available in the medical record it does not need to be repeated. HLA type may also be obtained from a frozen sample.
[5] Apheresis sample is recorded on "Visit 06A" tracking form even if the same day as Visit 06. If apheresis occurs on the same day as Visit 06, only draw 60 mL in EDTA tubes. Note that Visits 06 and 06A have overlapping, but different preferred visit windows. SST and EDTA tubes are drawn at Visit 06 even if apheresis is also done.
[6] Tubes from Visit 05 may be processed by Building 40 laboratories.
Visit windows: Schedule Visits 02A through 04 with respect to Day 0; Schedule Visits 04A through 08 with respect to Visit 04. The following Visit windows apply: 02A (±1 day), 02B, and 04A (+2 days); Visit 03 (±7 days); Visit 04 (±28 days); Visit 05 and 06 (±2 days); Visit 06A (-2, +7 days), Visit 07 (±7 days); Visit 08 (±14 days).

APPENDIX IV
TABLE FOR GRADING SEVERITY OF ADVERSE EVENTS

<u>Assessment of Causality Relationship of an Adverse Event (AE) to Study Vaccine</u>:

The relationship between an AE and the vaccine will be assessed by the investigator on the basis of his or her clinical judgment and the definitions below.

- **Definitely Related**. The AE and administration of study agent are related in time, and a direct association can be demonstrated.
- **Probably Related**. The AE and administration of study agent are reasonably related in time, and the AE is more likely explained by study agent than other causes.
- **Possibly Related**. The AE and administration of study agent are reasonably related in time, but the AE can be explained equally well by causes other than study agent.
- **Not Related**. There is not a reasonable possibility that the AE is related to the study agent.

For purposes of preparing data reports in which AE attributions are limited to "**Related**" or "**Not Related**", in this protocol, the "Definitely, Probably and Possibly" attributions will be mapped to the "Related" category. The definitions that apply when these two categories alone are used are as follows:

- **Related** – There is a reasonable possibility that the AE may be related to the study agent.
- **Not Related** – There is not a reasonable possibility that the AE is related to the study agent.

Grading the Severity of Adverse Events:
The FDA Guidance for Industry (September 2007): "Toxicity Grading Scale for Healthy Adult and Adolescent Volunteers Enrolled in Preventive Vaccine Clinical Trials" is the basis for the severity grading of adverse events in this protocol. Several modifications were made to the table as follows:

- "Emergency room visit" is not automatically considered a life-threatening event; these words have been removed from any "grade 4' definition where they appear in the table copied from the guidance document.
- Any laboratory value shown as a "graded" value in the table that is within the institutional normal range will not be severity graded or recorded as an adverse event.
- When a gap in calculated values would have occurred, the higher grade was extended to include values greater than the lower grade.
- Severity grading for hemoglobin decrease on the basis of the magnitude of decrease from baseline is not applicable at the grade 1 level; only absolute hemoglobin will be used to define grade 1 decrease. Increases in hemoglobin are AEs only for values above the upper limit of normal and are graded by the systemic illness clinical criteria.
- Severity grading definition for Grade 4 local reaction to injectable product (Erythema/Redness and Induration/Swelling) included added text "requiring medical attention".
- 1 X ULN was removed from the definition for PT increase.
- Severity grading definition for hypotension includes added clarifications such that an asymptomatic low blood pressure reading is not an adverse event.

When not otherwise specified in the table, the following guidance will be used to assign a severity grade:

Grade 1 (Mild): No effect on activities of daily living
Grade 2 (Moderate): Some interference with activity not requiring medical intervention
Grade 3 (Severe): Prevents daily activity and requires medical intervention
Grade 4 (Life-threatening): Hospitalization; immediate medical intervention or therapy required to prevent death.
Grade 5 (Death): Death is assigned a Grade 5 severity.
Only the single adverse event that is assessed as the primary cause of death should be assigned "grade 5" severity.

Toxicity Grading Scale for Healthy Adult and Adolescent Volunteers Enrolled in Preventive Vaccine Clinical Trials
Modified from FDA Guidance - September 2007

A. Tables for Clinical Abnormalities

**Local Reaction to Injectable Product**	**Mild (Grade 1)**	**Moderate (Grade 2)**	**Severe (Grade 3)**	**Potentially Life Threatening (Grade 4)**
Pain	Does not interfere with activity	Repeated use of non-narcotic pain reliever > 24 hours or interferes with activity	Any use of narcotic pain reliever or prevents daily activity	Hospitalization
Tenderness	Mild discomfort to touch	Discomfort with movement	Significant discomfort at rest	Hospitalization
[1]Erythema/Redness	2.5 – 5 cm	5.1 – 10 cm	> 10 cm	Necrosis or exfoliative dermatitis requiring medical attention
[2] Induration/Swelling	2.5 – 5 cm and does not interfere with activity	5.1 – 10 cm or interferes with activity	> 10 cm or prevents daily activity	Necrosis requiring medical attention
**[3] Vital Signs**	**Mild (Grade 1)**	**Moderate (Grade 2)**	**Severe (Grade 3)**	**Potentially Life Threatening (Grade 4)**
[4] Fever (°C)<br>(°F)	38.0 – 38.4<br>100.4 – 101.1	38.5 – 38.9<br>101.2 – 102.0	39.0 – 40<br>102.1 – 104	> 40<br>> 104
Tachycardia - beats per minute	101 – 115	116 – 130	> 130	Hospitalization for arrhythmia
[5] Bradycardia - beats per Minute	50 – 54	45 – 49	< 45	Hospitalization for arrhythmia
Hypertension (systolic) - mm Hg	141 – 150	151 – 155	> 155	Hospitalization for malignant hypertension
Hypertension (diastolic) - mm Hg	91 – 95	96 – 100	> 100	Hospitalization for malignant hypertension
Hypotension (systolic) – mm Hg	85 – 89 and symptomatic	80 – 84 and symptomatic and requiring oral fluids	< 80 and symptomatic and requiring IV fluids	Hospitalization for hypotensive shock
Respiratory Rate – breaths per minute	17 – 20	21 – 25	> 25	Intubation

1. In addition to grading the measured local reaction at the greatest single diameter, the measurement should be recorded as a continuous variable.
2. Induration/Swelling should be evaluated and graded using the functional scale as well as the actual measurement.
3. Subject should be at rest for all vital sign measurements.
4. Oral temperature; no recent hot or cold beverages or smoking.
5. When resting heart rate is between 60 – 100 beats per minute. Use clinical judgment when characterizing Bradycardia among some healthy subject populations, for example, conditioned athletes.

**Systemic (General)**	**Mild (Grade 1)**	**Moderate (Grade 2)**	**Severe (Grade 3)**	**Potentially Life Threatening (Grade 4)**
Nausea/vomiting	No interference with activity or 1 – 2 episodes/24 hours	Some interference with activity or > 2 episodes/24 hours	Prevents daily activity, requires outpatient IV hydration	Hospitalization for hypotensive shock
Diarrhea	2 – 3 loose stools or < 400 gms/24 hours	4 – 5 stools or 400 – 800 gms/24 hours	6 or more watery stools or > 800gms/24 hours or requires outpatient IV hydration	Hospitalization
Headache	No interference with activity	Repeated use of non- narcotic pain reliever > 24 hours or some interference with activity	Significant; any use of narcotic pain reliever or prevents daily activity	Hospitalization
Fatigue	No interference with activity	Some interference with activity	Significant; prevents daily activity	Hospitalization
Myalgia	No interference with activity	Some interference with activity	Significant; prevents daily activity	Hospitalization

**Systemic Illness**	**Mild (Grade 1)**	**Moderate (Grade 2)**	**Severe (Grade 3)**	**Potentially Life Threatening (Grade 4)**
Illness or clinical adverse event (as defined according to applicable regulations)	No interference with activity	Some interference with activity not requiring medical intervention	Prevents daily activity and requires medical intervention	Hospitalization

B. Tables for Laboratory Abnormalities

**Serum ***	**Mild (Grade 1)**	**Moderate (Grade 2)**	**Severe (Grade 3)**	**Potentially Life Threatening (Grade 4)**
Sodium – Hyponatremia mEq/L	132 – 134	130 – 131	125 – 129	< 125
Sodium – Hypernatremia mEq/L	144 – 145	146 – 147	148 – 150	> 150
Potassium – Hyperkalemia mEq/L	5.1 – 5.2	5.3 – 5.4	5.5 – 5.6	> 5.6
Potassium – Hypokalemia mEq/L	3.5 – 3.6	3.3 – 3.4	3.1 – 3.2	< 3.1
Glucose – Hypoglycemia mg/dL	65 – 69	55 – 64	45 – 54	< 45
Glucose – Hyperglycemia<br>Fasting – mg/dL<br>Random – mg/dL	100 – 110<br>110 – 125	111 – 125<br>126 – 200	>125<br>>200	Insulin requirements or hyperosmolar coma
Blood Urea Nitrogen BUN mg/dL	23 – 26	27 – 31	> 31	Requires dialysis
Creatinine – mg/dL	1.5 – 1.7	1.8 – 2.0	2.1 – 2.5	> 2.5 or requires dialysis
Calcium – hypocalcemia mg/dL	8.0 – 8.4	7.5 – 7.9	7.0 – 7.4	< 7.0
Calcium – hypercalcemia mg/dL	10.5 – 11.0	11.1 – 11.5	11.6 – 12.0	> 12.0
Magnesium – hypomagnesemia mg/dL	1.3 – 1.5	1.1 – 1.2	0.9 – 1.0	< 0.9
Phosphorous – hypophosphatemia mg/dL	2.3 – 2.5	2.0 – 2.2	1.6 – 1.9	< 1.6
CPK – mg/dL	1.25–1.5 xULN**	>1.5 – 3.0 x ULN	>3.0 –10 x ULN	> 10 x ULN
Albumin – Hypoalbuminemia g/dL	2.8 – 3.1	2.5 – 2.7	< 2.5	--
Total Protein – Hypoproteinemia g/dL	5.5 – 6.0	5.0 – 5.4	< 5.0	--
Alkaline phosphate – increase by factor	1.1 – 2.0 x ULN	>2.0 – 3.0 x ULN	>3.0 – 10 x ULN	> 10 x ULN
Liver Function Tests –ALT, AST increase by factor	1.1 – 2.5 x ULN	>2.5 – 5.0 x ULN	>5.0 – 10 x ULN	> 10 x ULN
Bilirubin – when accompanied by any increase in Liver Function Test increase by factor	1.1 – 1.25 x ULN	>1.25 – 1.5 x ULN	>1.5 – 1.75 x ULN	> 1.75 x ULN
Bilirubin – when Liver Function Test is normal; increase by factor	1.1 – 1.5 x ULN	>1.5 – 2.0 x ULN	>2.0 – 3.0 x ULN	> 3.0 x ULN
Cholesterol	201 – 210	211 – 225	> 226	---
Pancreatic enzymes – amylase, lipase	1.1 – 1.5 x ULN	>1.5 – 2.0 x ULN	>2.0 – 5.0 x ULN	> 5.0 x ULN

* The laboratory values provided in the tables serve as guidelines and are dependent upon institutional normal parameters. Institutional normal reference ranges should be provided to demonstrate that they are appropriate.
**ULN" is the upper limit of the normal range.

**Hematology ***	**Mild (Grade 1)**	**Moderate (Grade 2)**	**Severe (Grade 3)**	**Potentially Life Threatening (Grade 4)**
Hemoglobin (Female) - gm/dL	11.0 – 12.0	9.5 – 10.9	8.0 – 9.4	< 8.0
Hemoglobin (Female) decrease from baseline value - gm/dL	not applicable	1.6 – 2.0	2.1 – 5.0	> 5.0
Hemoglobin (Male) - gm/dL	12.5 – 13.5	10.5 – 12.4	8.5 – 10.4	< 8.5
Hemoglobin (Male) decrease from baseline value – gm/dL	not applicable	1.6 – 2.0	2.1 – 5.0	> 5.0
WBC Increase - cell/mm^3	10,800 – 15,000	15,001 – 20,000	20,001 – 25, 000	> 25,000
WBC Decrease - cell/mm^3	2,500 – 3,500	1,500 – 2,499	1,000 – 1,499	< 1,000
Lymphocytes Decrease - cell/mm^3	750 – 1,000	500 – 749	250 – 499	< 250
Neutrophils Decrease - cell/mm^3	1,500 – 2,000	1,000 – 1,499	500 – 999	< 500
Eosinophils - cell/mm^3	650 – 1500	1501 - 5000	> 5000	Hypereosinophilic
Platelets Decreased - cell/mm^3	125,000 – 140,000	100,000 – 124,000	25,000 – 99,000	< 25,000
PT – increase by factor (prothrombin time)	1.10 x ULN**	>1.1 – 1.2 x ULN	>1.2 – 1.25 x ULN	> 1.25 ULN
PTT – increase by factor (partial thromboplastin time)	1.10 – 1.20 x ULN	>1.2 – 1.4 x ULN	>1.4 – 1.5 x ULN	> 1.5 x ULN
Fibrinogen increase - mg/dL	400 – 500	501 – 600	> 600	--
Fibrinogen decrease - mg/dL	150 – 200	125 – 149	100 – 124	< 100 or associated with gross bleeding or disseminated intravascular coagulation (DIC)

* The laboratory values provided in the tables serve as guidelines and are dependent upon institutional normal parameters. Institutional normal reference ranges should be provided to demonstrate that they are appropriate.
**ULN" is the upper limit of the normal range.
