# Supplementary material for: An avian influenza H7 DNA priming vaccine is safe and immunogenic in a randomized phase I clinical trial
Source: NPJ Vaccines. 2017 Jun 1;2:15. doi: 10.1038/s41541-017-0016-6 (PMC5627236; doi:10.1038/s41541-017-0016-6)
Supplement: Supplementary file 2 — Supplementary Figure S1, Table S1, Table S2 [file 41541_2017_16_MOESM2_ESM.docx]

**Supplemental Figure Legends**

**Figure S1.** Induction of H7-specific antibodies following 3 different prime-boost regimens. HAI = hemagglutination inhibition assay. Geometric mean titers and 95% confidence intervals are shown at baseline (pre-boost), 2, 4, and 12 weeks following H7N9 boost vaccination (reported as baseline and study weeks 16, 18, 20 and 28). Group 1 received H7 DNA at day 0 and H7N9 MIV at week 16. Group 2 received both H7 DNA and H7N9 MIV prime at day 0 and H7N9 MIV at week 16. Group 3 received H7N9 MIV at day 0 and at week 16.

Figure S1

**Supplemental Table 1.** Baseline Characteristics Of Subjects

| **Characteristic** |  | **Group 1 (N=10)** | **Group 2 (N=10)** | **Group 3 (N=10)** | **Overall (N=30)** |
| --- | --- | --- | --- | --- | --- |
| N (%) | | | | | |
| GENDER | Male | 5 ( 50.0) | 3 ( 30.0) | 5 ( 50.0) | 13 ( 43.3) |
|  | Female | 5 ( 50.0) | 7 ( 70.0) | 5 ( 50.0) | 17 ( 56.7) |
| AGE (years) | Mean (S.D.) | 39.7 (8.8) | 31.5 ( 12) | 37.6 ( 11) | 36.3 ( 11) |
|  | Range | [27, 52] | [20, 53] | [22, 60] | [20, 60] |
| RACE | Asian | 0 ( 0.0) | 1 ( 10.0) | 1 ( 10.0) | 2 ( 6.7) |
|  | Black or African American | 3 ( 30.0) | 2 ( 20.0) | 0 ( 0.0) | 5 ( 16.7) |
|  | White | 7 ( 70.0) | 5 ( 50.0) | 9 ( 90.0) | 21 ( 70.0) |
|  | Multiracial | 0 ( 0.0) | 2 ( 20.0) | 0 ( 0.0) | 2 ( 6.7) |
| ETHNICITY | Non-Hispanic/Latino | 9 ( 90.0) | 10 (100.0) | 10 (100.0) | 29 ( 96.7) |
|  | Hispanic/Latino | 1 ( 10.0) | 0 ( 0.0) | 0 ( 0.0) | 1 ( 3.3) |
| BODY-MASS INDEX | Mean (S.D.) | 25.7 (5.4) | 28.0 (6.6) | 26.3 (3.7) | 26.6 (5.3) |
|  | Range | [19.9, 35.6] | [18.4, 39.3] | [20.5, 31.4] | [18.4, 39.3] |
| EDUCATION | High school graduate/GED | 1 ( 10.0) | 1 ( 10.0) | 1 ( 10.0) | 3 ( 10.0) |
|  | College/University | 5 ( 50.0) | 8 ( 80.0) | 4 ( 40.0) | 17 ( 56.7) |
|  | Advanced degree | 4 ( 40.0) | 1 ( 10.0) | 5 ( 50.0) | 10 ( 33.3) |

**Supplemental Table 2A.** Local Reactogenicity Following H7 DNA And H7N9 MIV Vaccinations In VRC 315

| **Symptoms** | **All H7 DNA Prime (N=20)** | **All H7N9 MIV Prime (N=20)** | **All H7N9 MIV Boost (N=28)** |
| --- | --- | --- | --- |
| PAIN/TENDERNESS |  |  |  |
| None | 3(15.0%) | 16(80.0%) | 22(78.6%) |
| Mild | 15(75.0%) | 4(20.0%) | 6(21.4%) |
| Moderate | 2(10.0%) | 0(0.0%) | 0(0.0%) |
| SWELLING |  |  |  |
| None | 20(100.0%) | 20(100.0%) | 28(100.0%) |
| Mild | 0(0.0%) | 0(0.0%) | 0(0.0%) |
| Moderate | 0(0.0%) | 0(0.0%) | 0(0.0%) |
| REDNESS |  |  |  |
| None | 20(100.0%) | 20(100.0%) | 28(100.0%) |
| Mild | 0(0.0%) | 0(0.0%) | 0(0.0%) |
| Moderate | 0(0.0%) | 0(0.0%) | 0(0.0%) |

**Supplemental Table 2B.** Systemic Reactogenicity Following H7 DNA and H7N9 MIV Vaccinations in VRC 315

| **Symptoms** | **Group 1**  **H7 DNA Prime**  **(N=10)** | **Group 2**  **H7 DNA Prime and H7N9 MIV Prime**  **(N=10)** | **Group 3**  **H7N9 MIV Prime**  **(N=10)** | **All Groups**  **MIV Boost**  **(N=28)** |
| --- | --- | --- | --- | --- |
| MALAISE |  |  |  |  |
| None | 8(80.0%) | 5(50.0%) | 10(100.0%) | 25(89.3%) |
| Mild | 2(20.0%) | 4(40.0%) | 0(0.0%) | 3(10.7%) |
| Moderate | 0(0.0%) | 1(10.0%) | 0(0.0%) | 0(0.0%) |
| MYALGIA |  |  |  |  |
| None | 10(100.0%) | 5(50.0%) | 9(90.0%) | 28(100.0%) |
| Mild | 0(0.0%) | 4(40.0%) | 1(10.0%) | 0(0.0%) |
| Moderate | 0(0.0%) | 1(10.0%) | 0(0.0%) | 0(0.0%) |
| HEADACHE |  |  |  |  |
| None | 8(80.0%) | 8(80.0%) | 8(80.0%) | 26(92.9%) |
| Mild | 2(20.0%) | 1(10.0%) | 2(20.0%) | 2(7.1%) |
| Moderate | 0(0.0%) | 1(10.0%) | 0(0.0%) | 0(0.0%) |
| CHILLS |  |  |  |  |
| None | 10(100.0%) | 9(90.0%) | 10(100.0%) | 28(100.0%) |
| Mild | 0(0.0%) | 0(0.0%) | 0(0.0%) | 0(0.0%) |
| Moderate | 0(0.0%) | 1(10.0%) | 0(0.0%) | 0(0.0%) |
| NAUSEA |  |  |  |  |
| None | 10(100.0%) | 9(90.0%) | 10(100.0%) | 28(100.0%) |
| Mild | 0(0.0%) | 1(10.0%) | 0(0.0%) | 0(0.0%) |
| JOINT PAIN |  |  |  |  |
| None | 10(100.0%) | 7(70.0%) | 10(100.0%) | 28(100.0%) |
| Mild | 0(0.0%) | 3(30.0%) | 0(0.0%) | 0(0.0%) |
| TEMPERATURE |  |  |  |  |
| None | 10(100.0%) | 10(100.0%) | 10(100.0%) | 28(100.0%) |
